# Tetrahedral [Sb(AuMe)_4_]^3−^ Occurring in Multimetallic Cluster Syntheses: About the Structure‐Directing Role of Methyl Groups

**DOI:** 10.1002/anie.202110526

**Published:** 2021-10-21

**Authors:** Fuxing Pan, Marcel Lukanowski, Florian Weigend, Stefanie Dehnen

**Affiliations:** ^1^ Fachbereich Chemie and Wissenschaftliches Zentrum für Materialwissenschaften (WZMW) Philipps-Universität Marburg Hans-Meerwein-Straße 4 35043 Marburg Germany

**Keywords:** antimony, DFT calculations, gold, intermetallic complex formation, X-ray diffraction

## Abstract

The anion of [K(crypt‐222)]_3_[Sb(AuMe)_4_]⋅py (**1**; crypt‐222=4,7,13,16,21,24‐hexaoxa‐1,10‐diazabicyclo[8.8.8]hexacosane; py=pyridine) represents a rare example of a homoleptic heavy p‐block metal atom being surrounded by four free‐standing transition metal complex fragments, and the third example for a corresponding Sb compound. In contrast to all reported complexes of this type, the transition metal atoms possess twofold coordination only, hence the complex as a whole does not exhibit significant steric shielding or further linkage of the metal atoms. This is reflected in a high flexibility, as confirmed by slight deviations from a tetrahedral coordination of the Sb atom in the crystal and soft vibrational modes. An alternative pyramidal conformer, observed for a related arsenic compound with terminal phosphine ligands, is apparently disfavored owing to electron correlation effects. The compound is formed in a reaction that in another solvent or at other reactant concentrations yields salts of ternary cluster anions. By a combined experimental and theoretical study of different reaction conditions and previously unidentified side‐products, we provide insight into multimetallic cluster synthesis reactions.

## Graphical abstract

[Sb(AuMe)_4_]^3−^ formed in a reaction of (Sn_2_Sb_2_)^2−^ with [AuMePPh_3_] that affords multimetallic clusters for other reactant ratios. A central Sb atom bridges four free‐standing {AuMe} units, which is unprecedented for main group metal complexes. Comprehensive DFT investigations explained the stability of the tetrahedral structure relative to a pyramidal one found in related molecules and shed light on the reaction space of said multimetallic species.

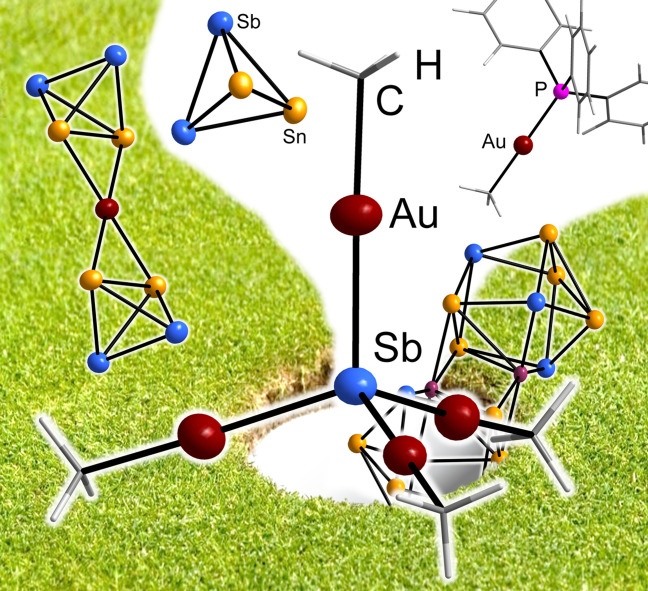

## Introduction

Multimetallic compounds have been in the focus of research groups across the world for a variety of reasons, ranging from the exploration of their mere accessibility or their structures and bonding, to their use as precursors to new intermetallic phases or in bond activation processes.[^1^, ^2^, ^3^, ^4^, ^5^, ^6^, ^7^] An elemental combination that attracted much attention is that of Sb and Au, due to relatively high electron affinities of both metals (Sb: 103.2 kJ mol^−1^, Au: 222.8 kJ mol^−1^), thus allowing for different polarization of the bond.^[8]^

One class of respective compounds comprises neutral or cationic complexes with essentially isolated Sb–Au contacts.[^9^, ^10^, ^11^, ^12^, ^13^, ^14^] In [((*o*‐(Ph_2_P)C_6_H_4_)_3_SbAuCl], for instance, the Sb–Au bond undergoes a reversible umpolung when being transferred to [((*o*‐(Ph_2_P)C_6_H_4_)_3_SbCl_2_AuCl];^[9a]^ this way, the typical Sb→Au σ‐donor interaction is strengthened through a reverse d(Au)→σ*(Sb–R) donation. Further examples of Sb–Au‐based complexes are the Z‐type dication [((*o*‐(Ph_2_P)C_6_H_4_)_2_(*o*‐Ph_2_PO)C_6_H_4_)SbAuCl]^2+^,^[9b]^ the T‐shaped 14‐e^−^ stiboranyl‐gold complex [(1,8‐naphthalene‐diyl)_2_(Ph_2_Sb)Au],^[9c]^ [Au(IPr)(Ar^NN^E)]^+^ with an N,C,N pincer‐type ligand Ar^NN^ chelating the Sb atom,^[10]^ or [Au_2_{(Ph_2_Sb)_2_O}_3_]^2+^ based on a central {Au_2_Sb_6_} unit.^[11]^ Moreover, two complexes were reported that comprise a tetrahedral {AuSb_4_} unit, [Au(SbPh_3_)_4_]^+^ and [(*p*‐tolyl)_2_Cl_2_Sb]Au[Sb(*p*‐tolyl)_3_]_3_.[^12^, ^13^, ^14^]

Another class of compounds exhibiting Sb–Au bonds are clusters in which the metal atoms are intensely interlinked. The cluster molecules are either protected by organic groups, as in [Au_8_(Et_3_P)_6_(SbPh)_2_(SbPh_2_)_4_],^[15]^ or stabilized by counterions, like [Sb_3_Au_3_Sb_3_]^3−^,^[16]^ [Au_2_Sb_16_]^4−^,^[17]^ and [(Tt_5_Sb_3_Au)_2_]^4−^ (Tt=Sn, Pb).^[18]^ Such ligand‐free multimetallic anions belong to the family of Zintl clusters that can be accessed by reacting homo‐ or heteroatomic p‐block metal anions with d‐block or f‐block metal compounds.^[7]^

In this context, reactions of *x* [K(crypt‐222)]_2_(Sn_2_Sb_2_)⋅en with *y* [AuMePPh_3_] were reported to yield [K(crypt‐222)]_3_[(Sn_2_Sb_2_)_2_Au] (**A**)[^19^, ^20^] in en (*x*:*y*=2:1) or [K(crypt‐222)]_4_[(Sn_5_Sb_3_Au)_2_]⋅2 py (**B**)^[18]^ in pyridine (*x*:*y*=1:2) (crypt‐222=4,7,13,16,21,24‐hexaoxa‐1,10‐diazabicyclo[8.8.8]‐hexacosane, en=ethane‐1,2‐diamine; py=pyridine), yet the underlying reactions are only poorly understood. The formation of the anion in **A**, [{(Sn_2_Sb_2_)^2−^}_2_Au^+^]^3−^, at first glance shows no sign of redox chemistry in comparison with the reactants. However, the anion in **B**, [{(Sn_5_Sb_3_)^3−^Au^+^}_2_]^4−^, is overall oxidized relative to the (Sn_2_Sb_2_)^2−^ anion—and in both cases, the gold complex apparently released not only the PPh_3_ groups, but also the (CH_3_)^−^ ligands. For this, it is very obvious that side reactions take place that have not yet been elucidated.

We were interested to understand these reactions better and therefore changed the ratio of the reactants [K(crypt‐222)]_2_(Sn_2_Sb_2_)⋅en and [AuMePPh_3_] from 2:1 or 1:1 to 1:2.2, and also searched for side‐products that are likely to form. This way, we obtained [K(crypt‐222)]_3_[Sb(AuMe)_4_]⋅py (**1**), the anion of which represents a very uncommon metal complex. Being based on an {SbAu_4_} core, thus inverse to the {AuSb_4_} core mentioned above, it is a very rare example of a heavy pnictogen atom being surrounded by free‐standing transition metal complex fragments.

Herein, we present the synthesis and crystal structure of **1**, report the electronic structure of the anion in comparison with related compounds, and comment on its formation reaction as opposed to the formation of the anionic clusters in **A** and **B**.

## Results and Discussion

**Scheme 1:**
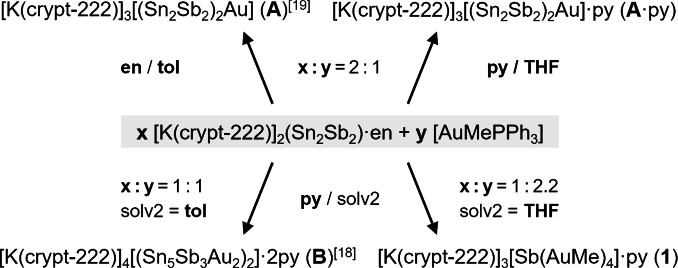
Illustration of the formation of compounds **1** (this work), **A**,^[19]^ **A**⋅py (this work), and **B** ^[18]^ by reactions of [K(crypt‐222)]_2_(Sn_2_Sb_2_)⋅en with [AuMePPh_3_] under different reaction conditions; en=ethane‐1,2‐diamine; py=pyridine; tol=toluene. Non‐crystalline (side‐)products are not given (see text for details).

Compound **1** is obtained from a reaction of 80 mg (0.058 mmol) of [K(crypt‐222)]_2_(Sn_2_Sb_2_)⋅en with 60 mg (0.125 mmol) of [AuMePPh_3_] in 3 mL of pyridine at room temperature. After stirring for 3 hours, filtration and layering with 3 mL of THF, the mixture is stored at 5 °C for four weeks. Crystals of **1** form in ≈16 % yield. Scheme 1 illustrates the synthesis of **1**, **A** (including a new solvate of it, **A**⋅py), and **B**, respectively. Noteworthy, other synthetic approaches to **1** starting out from K_3_Sb failed to work so far.

**Figure 1:**
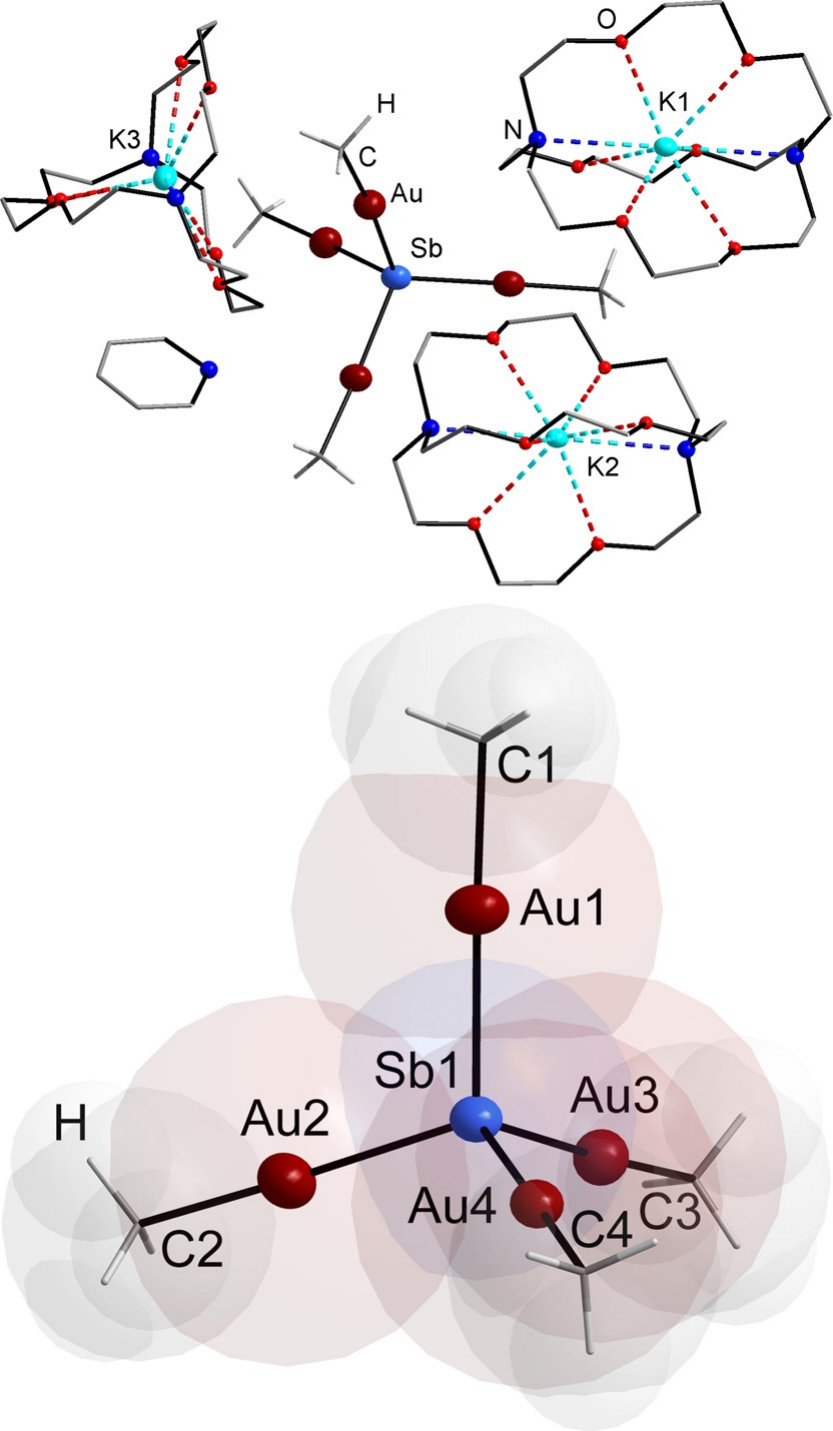
Single‐crystal structure of compound [K(crypt‐222)]_3_[Sb(AuMe)_4_]⋅py (**1**). Top: asymmetric unit, indicating the accommodation of the anionic complex between three cations and one solvent molecule. Bottom: structure of the anion with space‐filling model superimposed. Selected interatomic distances [Å] and angles [°]: Sb–Au 2.5923(9)–2.6080(10), Au–C 2.097(11)–2.121(9); Au–Sb–Au 101.78(3)–115.84(3), Sb–Au–C 174.4(4)–179.1(3).

Compound **1** crystallizes in the monoclinic space group *P*2_1_/*c* with four formula units in the unit cell. Figure 1 shows the structure of compound **1** and the anion in it in the crystal, as determined by means of single‐crystal X‐ray diffraction.

In the anion of **1**, a central μ^4^‐Sb atom possesses an idealized tetrahedral environment by the linearly coordinated Au atoms of four {AuMe} complex fragments. However, the Au–Sb1–Au angles actually indicate a certain degree of distortion. Quantum chemical calculations (see below) rationalize that this is due soft vibrational modes, and thus a high flexibility of the molecule as a whole that adopts a shape that fits best to the cation environment.

**Figure 2:**
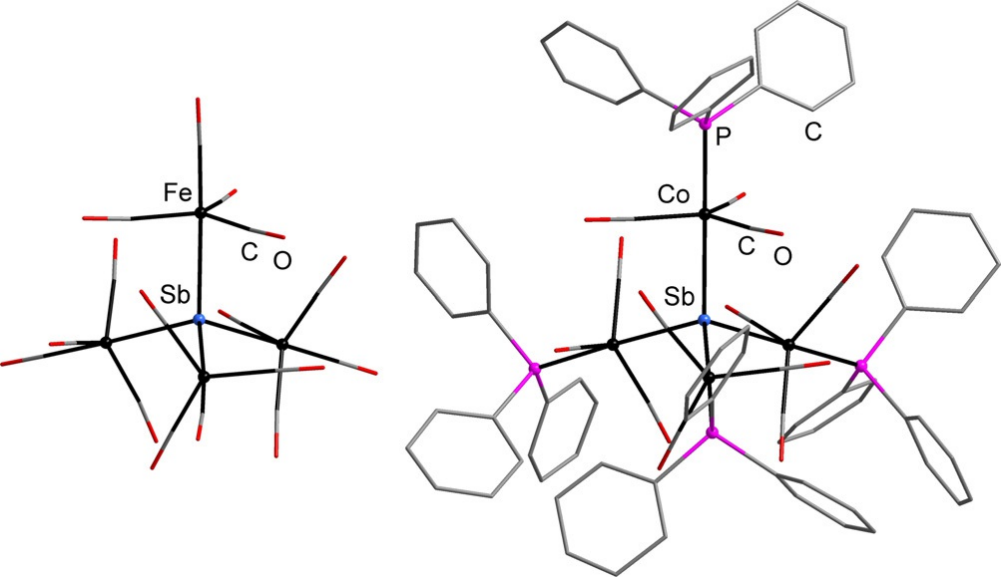
Molecular structures of the two literature‐known complexes of the type [Sb(ML_*n*_)_4_]^*q*^ with free‐standing metal complex fragments {ML_*n*_} μ^4^‐bridged by the Sb atom, [Sb{Fe(CO)_4_}_4_]^3−^ (left)^[21]^ and [Sb{Co(CO)_3_PPh_3_}_4_]^+^ (right).^[22]^

Indeed, only very few compounds have been described to date, in which a heavy (thus large) pnictogen atom acts as a μ^4^‐bridge to four free‐standing transition metal complex fragments that do not exhibit any further linkage of central and attached metal atoms, or among the attached metals atoms in cluster architectures. For Sb as the central atom, this was reported for two complexes only, [Sb{Fe(CO)_4_}_4_]^3−^ ^[21]^ and [Sb{Co(CO)_3_PPh_3_}_4_]^+^,^[22]^ according to the CCDC data base (7/2021; see Figure 2), and besides one P‐centered and three N‐centered cations,[^23^, ^24^, ^25^] this was not observed with Au complex fragments for any pnictogen atom so far. The rare observation of such complexes seemed to be due to rather labile bonding, which required kinetic stabilization by bulky complex fragments bearing four CO ligands or three CO and one PPh_3_ ligands, respectively. Beyond this background, the formation and crystallization of compound **1**, with little steric shielding, is even more remarkable.

**Figure 3:**
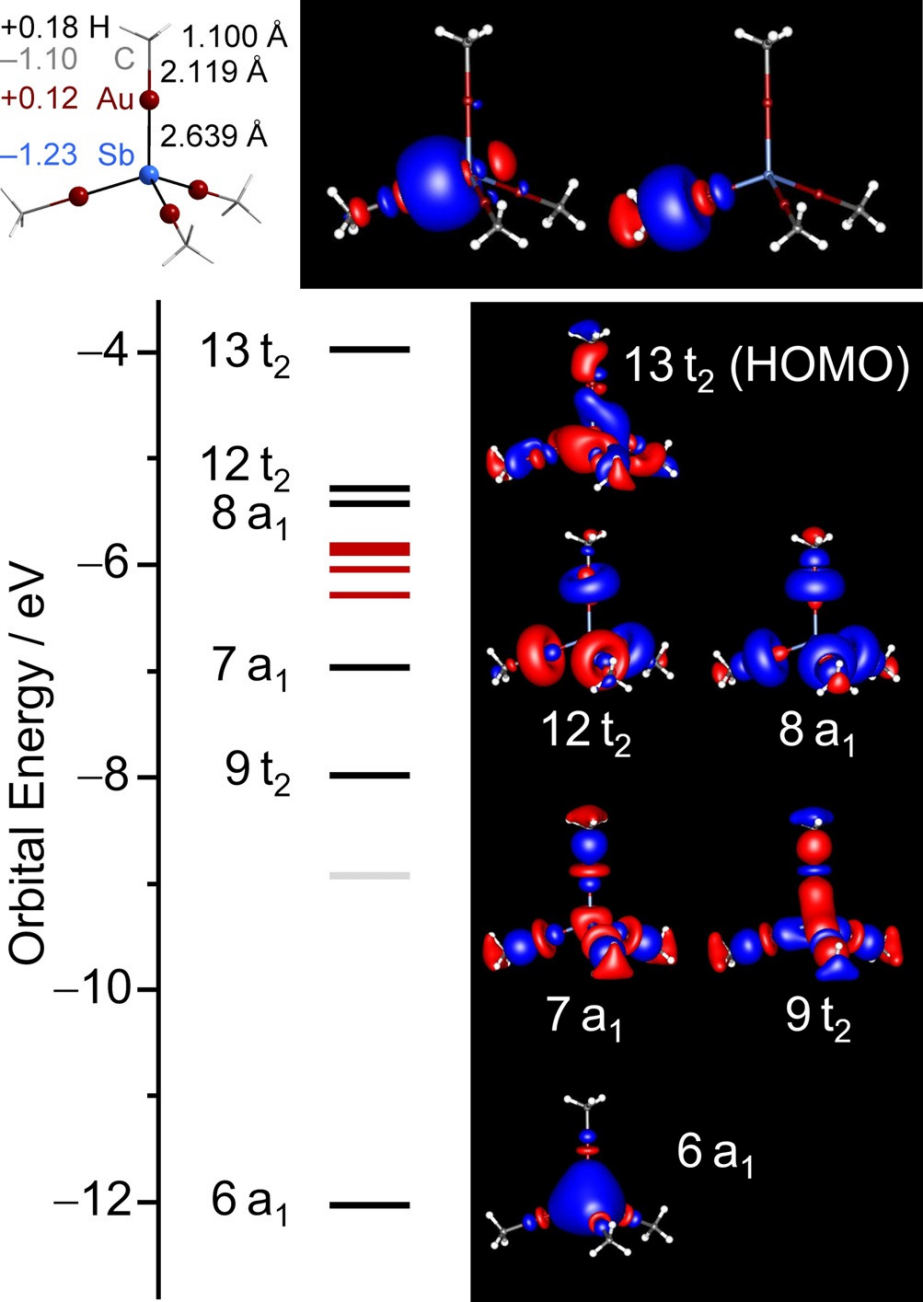
Top left: calculated structure of the [Sb(AuMe)_4_]^3−^ anion (*T*_d_ symmetry), with natural charges (left hand side) and optimized bond lengths (right hand side). Top center and right: localized molecular orbitals (LMOs) with highest energy expectation values, representing the Sb–Au and the Au–C bond. Bottom left: MO scheme of [Sb(AuMe)_4_]^3−^ (*T*_d_ symmetry); MOs being dominated by d(Au) are marked in red, those being located mainly at C and H are marked in light gray. Bottom right: molecular orbitals (MOs) representing the bonding between Sb and Au; only one column of the triply‐degenerate irreducible representations 13t_2_ and 9t_2_ are shown. Contours are plotted at ±0.03 a.u.

To analyze the bonding situation in the anion in **1**, we carried out DFT calculations (functional TPSS,^[26]^ basis set dhf‐TZVP,^[27]^ together with corresponding effective core potentials;^[28]^ charge compensation with the conductor‐like screening model^[29]^) with the program system turbomole. ^[30]^ The molecular structure was accurately reproduced, so we inspected the molecular orbitals (MOs) that were additionally localized into localized molecular orbitals (LMOs) by application of Boys’ method,^[31]^ and we calculated natural charges by means of natural population analyses (NPA).^[32]^ Results of these analyses are presented in Figure 3, along with selected LMOs. The MO diagram of **1**, is shown in Figure 3 and discussed below. Further plots of canonical MOs and LMOs are provided in Figures S35 and S36, respectively, detailed lists of contributions of atomic to molecular orbitals for **1** and related compounds are listed in Tables S7–S10.

While the overall dipole moment of the molecule is zero owing to its perfect *T*_d_ symmetry in the calculation, the local bonds are not unpolar. The natural charges (given in the atoms’ colors in Figure 3, top left) of the Sb or Au atoms are similar to those of the C or H atoms, respectively (cf. calculations of [K(crypt‐222)]^+^ using the same methods, yield natural charges of K, O, N, C, and H atoms as +0.88, −0.53, −0.43, −0.07⋅⋅⋅−0.23, and +0.17⋅⋅⋅+0.21, respectively). It is thus reasonable to assume that the Sb–Au bonds possesses a similar polarity as the C–H bonds. Correspondingly, the LMOs representing Sb–Au and Au–C bonds (Figure 3, top center and right) indicate a polarization towards the more electronegative elements Sb and C, as expected. In detail, the bonding can be classified as follows: The HOMO (13t_2_) mainly comprises contributions of 5p and 6p atomic orbitals (AOs) from Sb and Au atoms, respectively. Further bonding orbitals are 9t_2_, mainly composed of 5p (Sb) and 5d (Au), 8a_1_, mainly composed of 6p (Au) and 2p (C), and 7a_1_, mainly composed of 5d (Au) and 2p (C). A minor contribution from 6p (Au) is also observed for 7a_1_, which mainly represents the Sb 5s AO though. Hence, with the $5d_{z^{2}}$ AO of the Au atoms acting as an “extension bar” without changing the phase of the wave function, the overall situation is thus reminiscent of the CH_4_ molecule itself or the isoelectronic (NH_4_)^+^, which is in agreement with the anion's relative stability that at least suffices for its formation and crystallization as the ionic compound **1**.

Calculation of the vibrational modes (Figure S37 and Table S6) indicate that deformations of the {SbAu_4_} unit occur at wavenumbers below 200 cm^−1^. Symmetric and asymmetric stretching modes of the complex are calculated between 450 and 470 cm^−1^. All higher‐energy modes are assigned to vibrations of the ‐CH_3_ groups. The findings confirm the structural flexibility of the molecule mentioned above, and they also suggest that both the Sb–Au bonds and the Au–C bonds are readily broken. This is reflected by the ESI(−) mass spectrum of a fresh solution of single‐crystals of compound **1** (Figure S5). The most (and essentially only) prominent signal originates from Au^−^ (196.9681 *m*/*z* Figure S6), with a less intensive signal stemming from (AuMe)^−^ (211.9917 *m*/*z*, Figure S7), while the heterometallic complex is only reflected in two fragments, [Sb(AuCH_3_)_2_]^−^ (544.8776 *m*/*z*, Figure S8) and [{K(crypt‐222)}{SbAu_4_(CH_3_)_2_}]^−^ (154.0415 *m*/*z*, Figure S10). The latter are observed with lower relative abundances than the obvious decomposition product Sb_7_^−^ (852.3913 *m*/*z*, Figure S9). These findings reflect that the Sb–Au bonds in **1** are free‐standing and unsupported by any further interactions. Hence, although the bonds are shorter (2.5923(9)–2.6080(10) Å) and thus virtually stronger than those of the reported complexes [((*o*‐(Ph_2_P)C_6_H_4_)_2_(*o*‐Ph_2_PO)C_6_H_4_)SbAuCl]^2+^ (2.627 Å),^[9b]^ [Au(SbPh_3_)_4_]^+^ (2.656–2.658 Å),^[13]^ [((*o*‐(Ph_2_P)C_6_H_4_)_2_SbCl_2_AuCl] (2.7086 Å),^[9a]^ or [(1,8‐naphthalene‐diyl)_2_(Ph_2_Sb)Au] (2.762 Å),^[9c]^ the molecule as a whole is very fragile.

**Figure 4:**
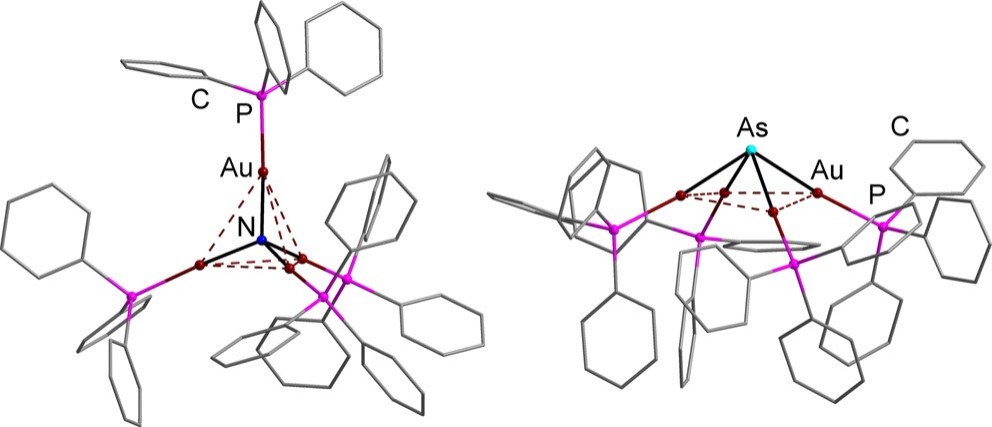
Molecular structures of the cationic complexes [N(AuPPh_3_)_4_]^+^ (bottom left)^[24a]^ and [As(AuPPh_3_)_4_]^+^ (bottom right)^[34]^ with tetrahedral or pyramidal structures, respectively.

Some years ago, a series of related, yet cationic, complexes [Pn(AuPH_3_)_4_]^+^ (Pn=N, P, As, Sb), were studied by DFT and ab initio calculations.^[33]^ The study was done to rationalize the experimental finding of “gilded ammonium” [N(AuPPh_3_)_4_]^+^ to adopt *T*_d_ symmetry,^[24]^ like the (NH_4_)^+^ cation itself, and the experimentally found structure of the heavier homologue [As(AuPPh_3_)_4_]^+^,^[34]^ which possesses *C*_4*v*_ symmetry (Figure 4). The different structures were put down to the existence of aurophilic interactions in the tetrahedral complex, which the authors found weakened as the central atom's radius is increased. Consequently, it was predicted that (unknown) [P(AuPH_3_)_4_]^+^ or [Sb(AuPH_3_)_4_]^+^ should adopt *T*_d_ or *C*_4*v*_ symmetries, respectively.^[33]^

**Figure 5:**
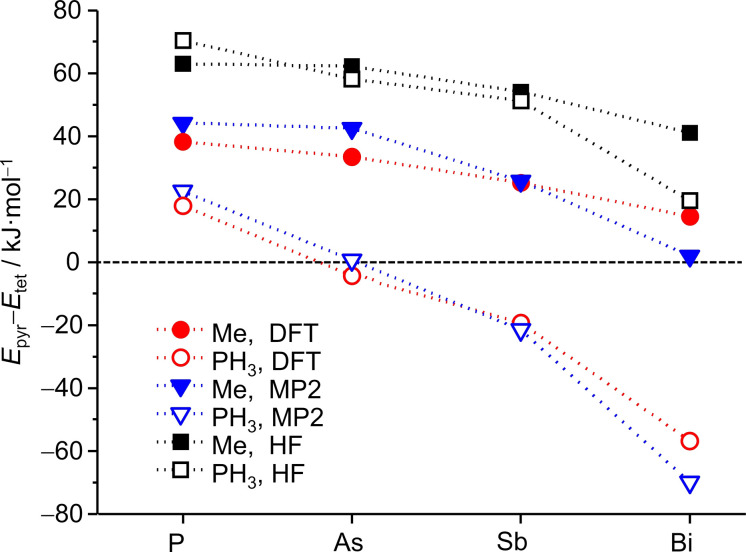
Energy difference of pyramidal and tetrahedral structures of [Pn(AuMe)_4_]^3−^ and [Pn(AuPH_3_)_4_]^+^ with the TPSS functional (DFT), SCS‐MP2 (MP2) and Hartree–Fock (HF); negative values indicate the preference of the pyramidal structure.

As this prediction obviously does not hold for the tetrahedral anion in **1**, [Sb(AuMe)_4_]^3−^, we repeated these calculations and compared the results with the series of cluster homologous to the anion in **1** at DFT level (TPSS/dhf‐TZVP), MP2 level (SCS‐MP2^[35]^/dhf‐TZVPP, with default parameters) and Hartree–Fock level (HF/dhf‐TZVP), see Figure 5. Results at DFT level (red color) and at MP2 level (blue color) are very similar and show the following trends. For Me substituents, the tetrahedral structure is generally preferred (with decreasing tendency from P to Bi), for PH_3_, this is true only for P as central atom, whereas for Sb‐ and Bi‐centered complexes, the pyramidal structure would indeed be favored, and for As, both structures are very similar in energy. Hence, the presence of a (inversely polarized) methyl group causes the structure of **1** to be tetrahedral. For the central atom being N, the pyramidal structure shows an imaginary mode. Distortion along this mode lowers the symmetry to *C*_2*v*_, and subsequent optimization results in the tetrahedral structure. This compound is thus not shown in Figure 5. At Hartree–Fock level (black color), the tetrahedral structure is clearly preferred throughout. Thus, we may conclude that the preference of the pyramidal structure is due to electron correlation which is neglected at Hartree–Fock level, but included both in the DFT and the MP2 treatment.

It is stressed that this is a correlation between *all* valence electrons, and not just a dispersive interaction between the d electrons of the Au atoms. This becomes clear when comparing the Au–Pn–Au angle in the pyramidal structure for three different calculations, exemplarily discussed here for [Sb(AuPH_3_)_4_)]^+^: at HF level, it amounts to 115.2°, at MP2 level to 103.3°. In a third calculation we correlated only the Au(d) orbitals which are energetically reasonably well separated from the other orbitals, see Table S11. This yields four d orbitals per Au atom, the fifth is involved in the bonds to the neighbors. In this way, one gets 114.5°, which is very close to the HF result. If there had been a notable difference, this had indicated that the electrons in the four d orbitals located at the Au atom had undergone dispersive interactions. As this is not the case, the result shows that dispersive interactions of Au(d) electrons (often denoted as “aurophilic”) are not structure‐determining here. We finally note that the tetrahedral and the pyramidal structures are separated by energy barriers that are estimated to be in the range of 32 kJ mol^−1^ ([Bi(AuPH_3_)_4_]^+^) to 45 kJ mol^−1^ ([Sb(AuMe)_4_]^3−^) higher than the tetrahedral structure (calculated at DFT level with a nudge‐band type path optimization tool^[36]^). Cartesian coordinates of all calculated structures are deposited in the Supporting Information in the file “Structures‐Calc.xyz”.

**Scheme 2:**
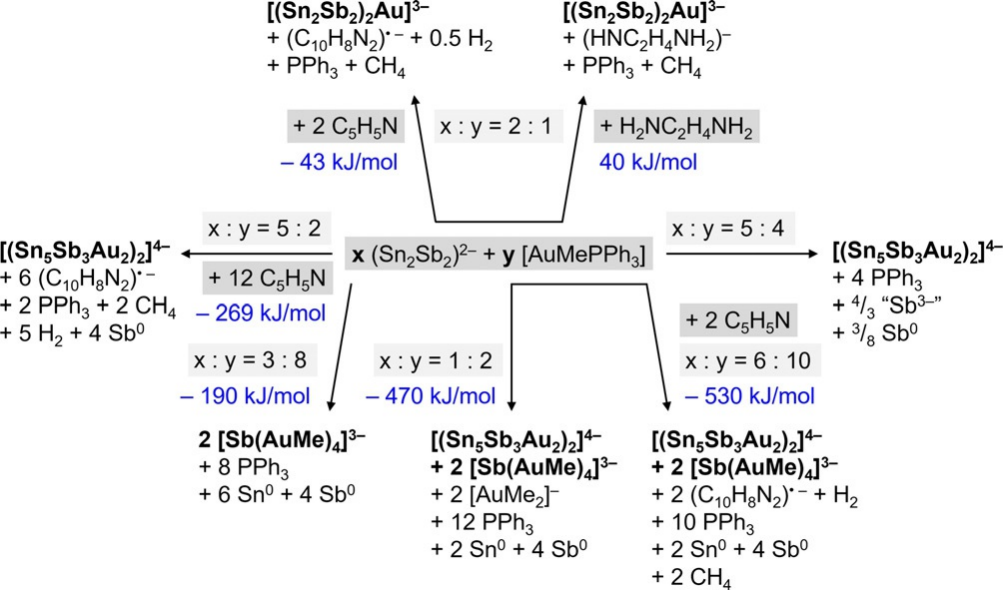
Suggestion of reactions to take place at different stoichiometries of (Sn_2_Sb_2_)^2−^ and [AuMePPh_3_], and in different solvents, to form the ternary anions [(Sn_2_Sb_2_)_2_Au)]^3−^ (in **A**, top), [(Sn_5_Sb_3_Au_2_)_2_]^4−^ (in **B**, center and bottom), or [Sb(AuMe)_4_]^3−^ (in **1**, bottom), respectively. Note that all mentioned side‐products were experimentally proven to form (“Sb^3−^” in **1** as soon as enough {AuMe} is available), except for the 4,4′‐bipyridyl radical anion (C_10_H_8_N_2_)^.−^ and (H_2_NCH_2_CH_2_NH)^−^; these anions, however, were mentioned as side‐products of Zintl cluster formation in previous work.[^7b^, ^37^]

As mentioned above, the formation of the anions in compounds **A**, **B**, and **1** require that additional side products are formed, which have so far not been identified for any of these reactions.[^18^, ^19^, ^20^] We therefore drew up possible reaction schemes (see Scheme 2) for the different reactant stoichiometries and solvents. The formation of the side products indicated in these reaction schemes was rationalized by means of energy‐dispersive X‐ray (EDX) spectroscopy and powder‐X‐ray diffraction (PXRD) of the solid precipitate, by ^31^P‐NMR spectroscopy and electrospray ionization mass spectrometry (ESI‐MS) of the reactive solutions, by detection of CH_4_ gas using gas chromatography‐mass spectrometry (GCMS), and by detection of H_2_ gas with the Pd/MoO_3_ method. In addition, quantum chemical calculations of corresponding reaction energies (Tables S12 and S13) suggested that the given reaction schemes are plausible.

In agreement with the experimental findings, metallic precipitates are observed in most of the reactions; for the formation reaction yielding compound **1**, thus in an ≈1:2 ratio of the reactants, the precipitate was confirmed to contain (crystalline) Sb and Sn metal (Table S5, Figures S13–S15). We could additionally show by ^31^P‐NMR spectroscopy that PPh_3_ is released under conditions that lead to the formation of all products (Figures S16–S20). The formation of [AuMe_2_]^−^ ^[38]^ was detected in the ESI mass spectrum of reaction solution with a 1:2 ratio of the reactants (Figures S22 and S25), and GCMS studies served to prove the formation of CH_4_ (Figures S28–S31) under the same conditions (in pyridine). The formation and release of H_2_ gas (Figures S32 and S33) was indeed only detected for the 1:2 reactant ratio in pyridine (leading to the formation of **1** or **B**), while no H_2_ is released for the 2:1 ratio in en (leading to the formation of **A**)—in agreement with the reaction schemes shown above.

The calculated reaction energies are to be taken with a grain of salt, as (a) for reactions that indicate the formation of metals, the (experimental) values of the atomization energies needed to be taken into consideration, and as (b) calculation of gas phase species instead of crystalline compounds neglects the beneficial lattice energy, which might overcompensate small energy differences, such as ±40 kJ mol^−1^. Yet the numbers indicate that most of the reactions are overall favorable and that the assumption of a key reactive role of the solvent in such processes seems to be plausible.

## Conclusion

In summary, we reported a new product in the reaction space of an Sn–Sb‐based binary Zintl anion and an Au complex yielding multimetallic products. The anion of the title compound [K(crypt‐222)]_3_[Sb(AuMe)_4_]⋅py (**1**) comprises a central Sb atom serving as a μ^4^‐bridge to four free‐standing {AuMe} units. It is thus a rare example of complexes based on an {SbM_4_} unit without further linkage of the transition metal M atoms or further bridging of the Sb and M atoms. Comprehensive quantum chemical studies showed that electron correlation effects stabilize this labile tetrahedral structure, while a related species with Pn=As and PPh_3_ ligands, instead of methyl substituents, was reported to adopt a pyramidal shape. A combination of experimental and quantum chemical investigations served to suggest possible reaction schemes that yield three different anions, and to rationalize that their occurrence is mainly controlled by the ratio of the used reactants, (Sn_2_Sb_2_)^2−^ and [AuMePPh_3_], and the solvent. Although mechanistic details cannot be gained from these complex reaction systems‐since kinetic studies and reasonable in situ monitoring fail owing to the lack of key spectroscopic handles and due to the difficulties in drawing conclusions from time‐dependent mass spectra‐we were able to shed some light on the still widely unexplored landscape of multimetallic cluster synthesis and on the nature of the uncommon compounds isolated from it. We are confident that this knowledge can be transferred to the synthesis of many similar systems in the future.

## Conflict of interest

The authors declare no conflict of interest.

## Supporting information

As a service to our authors and readers, this journal provides supporting information supplied by the authors. Such materials are peer reviewed and may be re‐organized for online delivery, but are not copy‐edited or typeset. Technical support issues arising from supporting information (other than missing files) should be addressed to the authors.

Supporting Information

Supporting Information

Supporting Information
