# Supplementary material for: Tetrahedral [Sb(AuMe)4]3− Occurring in Multimetallic Cluster Syntheses: About the Structure‐Directing Role of Methyl Groups
Source: Angew Chem Int Ed Engl. 2021 Oct 21;60(47):25042–7. doi: 10.1002/anie.202110526 (PMC9298313; doi:10.1002/anie.202110526)
Supplement: Supplementary file 2 — Supporting Information [file ANIE-60-25042-s003.pdf]

Angewandte Chemie
Eine Zeitschrift der Gesellschaft Deutscher Chemiker

Supporting Information

Tetrahedral $[Sb(AuMe)_4]^{3-}$ Occurring in Multimetallic Cluster Syntheses: About the Structure-Directing Role of Methyl Groups

*Fuxing Pan, Marcel Lukanowski, Florian Weigend, and Stefanie Dehnen**

anie_202110526_sm_miscellaneous_information.pdf
anie_202110526_sm_cif.zip
anie_202110526_sm_SI.xyz

1 Synthesis Details

1.1 General

All manipulations and reactions were performed under dry Ar atmosphere using standard Schlenk or glovebox techniques. All solvents were dried and freshly distilled prior to use; crypt-222[1] (Merck) was dried in vacuo for at least 18 hours. [K(crypt-222)]$_2$(Sn$_2$Sb$_2$)·en (en = ethane-1,2-diamine) were prepared according to literature.[2] [AuMePPh$_3$] was commercially available from Aldrich. Samples were shielded from ambient light throughout cluster syntheses.

1.2 Synthesis of [K(crypt-222)]$_3$[Sb(AuMe)$_4$]·py (1)

80 mg (0.058 mmol) of [K(crypt-222)]$_2$(Sn$_2$Sb$_2$)·en and 60 mg (0.125 mmol) of [AuMePPh$_3$] were combined in a Schlenk tube and dissolved in pyridine (py; 3 mL). The initially dark red solution slowly changed to yellow, along with black precipitation. The reaction mixture was allowed to stir for 3 hours. The resulting yellow solution was filtered through a standard glass frit, carefully layered with THF (3 mL), and stored for crystallization at 5 °C. After 25 days, plate-like yellow crystals of compound **1** formed at the wall of the Schlenk tube in appr. 16 % yield. Figure S1 shows light-microscopic image of the crystals, which appear hexagons or platelets of other shapes. All tested crystals comprised (**1**). The high air sensitivity of the compound is visible by a fine black powder covering the surface of the crystals, which indicates beginning decomposition even under high-viscosity oil.

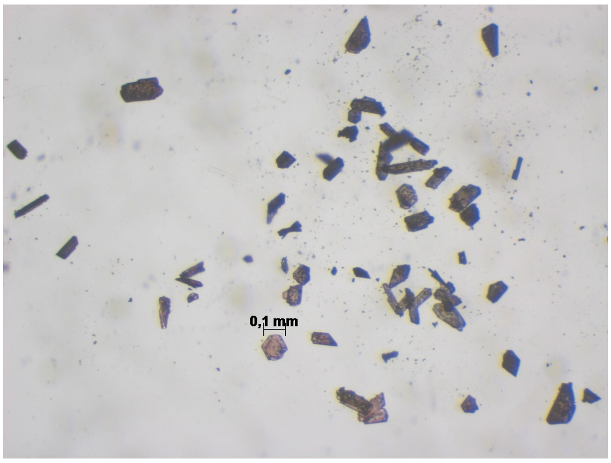

Figure S1. Crystal photograph of **1** taken under a light microscope. The surface of the crystals is covered with a fine black powder, which indicates beginning decomposition.

1.3 Alternative Synthesis of [K(crypt-222)]$_3$[(Sn$_2$Sb$_2$)$_2$Au] (A) from Ethane-1,2-Diamine, and Synthesis of [K(crypt-222)]$_3$[(Sn$_2$Sb$_2$)$_2$Au]·py (A·py) from Pyridine

Compound **A**, which was reported in ref. [3] to be obtained from a reaction of [K(crypt-222)]$_2$[Sn$_2$Sb$_2$]·en with [AuPhPPh$_3$], could also be isolated selectively from a reaction of [K(crypt-222)]$_2$(Sb$_2$Sn$_2$)·en with [AuMePPh$_3$] in en: 80 mg (0.058 mmol) of [K(crypt-222)]$_2$(Sn$_2$Sb$_2$)·en and 14 mg (0.029 mmol) of [AuMePPh$_3$] were combined in a Schlenk tube and dissolved in 3 mL of en. The reaction mixture was allowed to stir for 3 hours. The resulting dark red solution was filtered through a standard glass frit, carefully layered with 3 mL of toluene, and stored for crystallization at 5 °C. After 7 days, deep red plate-like crystals of compound **A** formed at the wall of the Schlenk tube in appr. 55 % yield (See Figure S2, left). A single-crystal X-ray measurement indicated the same crystal data as reported for CCDC 1044092.

If performing the reaction in pyridine (py) instead, 80 mg (0.058 mmol) of [K(crypt-222)]$_2$(Sn_2Sb_2)·en and 14 mg (0.029 mmol) of [AuMe(PPh_3)] were combined in a Schlenk tube and dissolved in 3 mL of py. The reaction mixture was allowed to stir for 3 hours. The resulting dark red solution was filtered through a standard glass frit, carefully layered with 3 mL of toluene, and stored for crystallization at 5 °C. After 10 days, dark red block-shaped crystals of compound **A**·py formed at the wall of the Schlenk tube in appr. 80 % yield (Figure S2, right).

According to the quantum chemical studies on the reactions (see Section 5, Table S8), the two approaches differ in the formation of H_2, which is observed in solvent en, while it is not observed in solvent py.

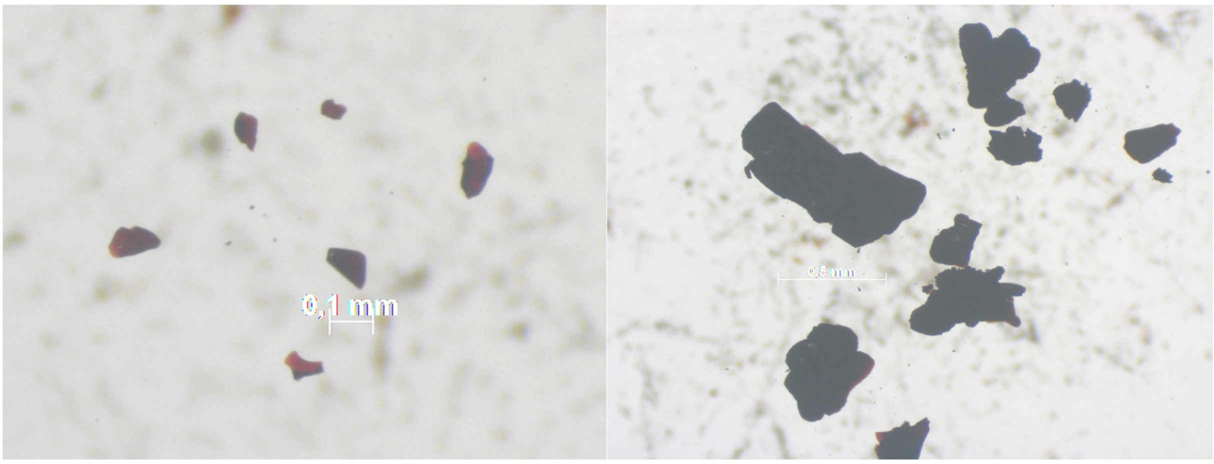

Figure S2. Crystal photographs of **A** (left) and **A**·py (right) taken under a light microscope.

2 Single-Crystal X-Ray Crystallography (SCXRD)

2.1 Single-Crystal X-ray Diffraction

The data for the X-ray structural analyses were collected at T = 100.0 K with Cu-Kα-radiation (λ = 0.71073 Å) on an area detector system Stoe StadiVari. The structure was solved by dual-space methods in SHELXT from SHELXL-2018/136,[4a] and refined by full matrix least-squares methods against F^2 with the SHELXL program.[4b] The relatively large tendency of the crypt-222 molecules for disorder in compound **1** required the application of restraints to the corresponding atoms; an alternative application of the back-Fourier-transform method did not produce more reliable results. Due to the moderate quality of the crystal data, the contribution of one heavily disordered solvent molecule to the overall structure factors was modelled by the back Fourier transform method by applying the SQUEEZE algorithm in the PLATON program package.[5] All hydrogen atoms were modelled as riding on calculated positions with isotropic displacement parameters U = 1.2 U_{eq} of the bonding partners. The CIF of compound **1** was deposited at the Cambridge Crystallographic Data Centre as CCDC 068423. A copy of the data can be obtained free of charge on application to CCDC, 12 Union Road, Cambridge CB2 1EZ, UK [fax.: (internat.) + 44 1223/336-033; e-mail: depos-it@ccdc.cam.ac.uk]. The crystal data and experimental parameters of the structure determinations are collected in Table S1. Supplementary views of the crystal structures are provided in Figures S2 and S3, created with Diamond 4.[6] Structural parameters are given in Tables S2 and S3.

Table S1. Crystal data and details of the structure determination of 1.

**Compound**	**1**	**A·py**
Empirical formula	$C_{63}H_{122}Au_4K_3N_7O_{18}Sb$	$C_{59}H_{111}AuK_3N_7O_{18}Sb_4Sn_4$
Formula weight, [g mol^{-1}]	2292.58	2482.57
Temperature/K	100	100
Crystal system	monoclinic	monoclinic
Space group	$P2_1$/c	$C2$/c
a/Å	11.921(2)	22.588(5)
b/Å	20.179(4)	14.406(3)
c/Å	36.075(7)	27.749(6)
α/°	90	90
β/°	97.35(3)	107.85(3)
γ/°	90	90
Volume/Å^3	8607(3)	8595(3)
Z	4	4
ρ_{calc}g/cm^3	1.769	1.919
μ/mm^{-1}	7.310	4.283
F(000)	4468.0	4792
Radiation	MoKα (λ= 0.71073)	MoKα (λ = 0.71073)
2Θ range for data collection/°	3.042 to 53.214	3.404 to 53.22
Index ranges	$-14 \leq h \leq 14$, $-24 \leq k \leq 11$, $-45 \leq l \leq 44$	$-28 \leq h \leq 26$, $-16 \leq k \leq 18$, $-34 \leq l \leq 31$
Reflections collected	106457	44970
Independent reflections	17668 [R_{int} = 0.0693, R_{sigma} = 0.0556]	8846 [R_{int} = 0.0680, R_{sigma} = 0.0477]
Data/restraints/parameters	17668/527/872	8846/0/436
Goodness-of-fit on F^2	0.939	0.986
Final R indexes [I>=2σ (I)]	R_1 = 0.0612, wR_2 = 0.1569	R_1 = 0.0600, wR_2 = 0.1598
Final R indexes [all data]	R_1 = 0.0837, wR_2 = 0.1732	R_1 = 0.0800, wR_2 = 0.1745
Largest diff. peak/hole / e Å^{-3}	1.33/-2.65	1.23/-2.27
CCDC deposition number	2068423	2075266

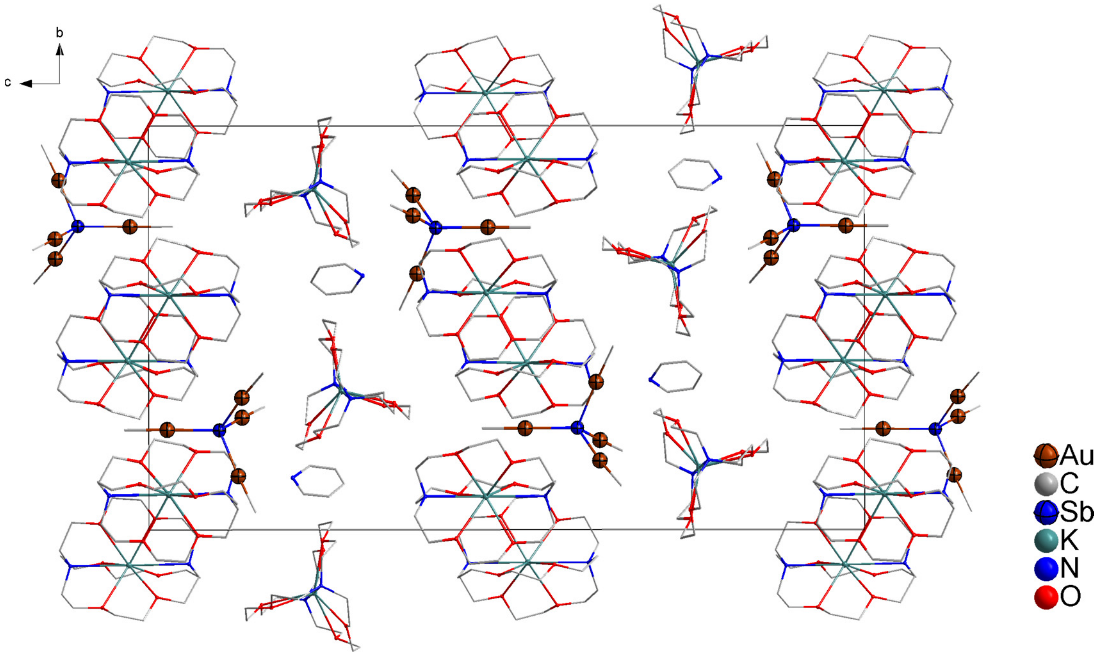

Figure S2. Unit cell view of compound **1**. Atoms are shown as thermal ellipsoids with 50% probability.

Table S2. Selected interatomic distances (in Å) and bond angles (in degrees) of the experimental and optimized structures of the anion in 1. The quantum chemical methods are detailed in Section 5.

Atoms	Distance (exp)	Distance (calc, T_d symmetry)
Sb1 – Au1	2.5923(9)	2.628
Sb1 – Au2	2.6045(11)	
Sb1 – Au3	2.6015(10)	
Sb1 – Au4	2.6080(10)	
Au1 – C1	2.097(11)	2.121
Au2 – C2	2.097(12)	
Au3 – C3	2.093(12)	
Au4 – C4	2.121(9)	
**Atoms**	**Angles (exp)**	**Angles (calc, T_d symmetry)**
Au1 – Sb1 – Au2	107.92(3)	109.47
Au1 – Sb1 – Au3	101.78(3)	
Au1 – Sb1 – Au4	112.04(3)	
Au2 – Sb1 – Au3	102.94(3)	
Au2 – Sb1 – Au4	115.07(4)	
Au3 – Sb1 – Au4	115.84(3)	
Sb1 – Au1 – C1	176.2(4)	
Sb1 – Au2 – C2	174.4(4)	
Sb1 – Au3 – C3	175.1(4)	
Sb1 – Au4 – C4	179.1(3)	

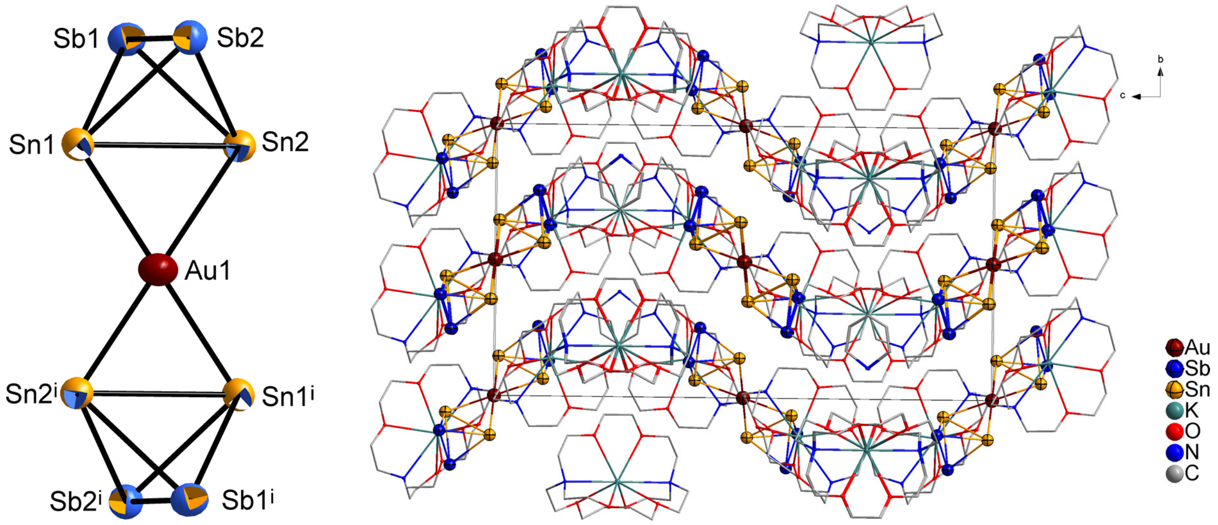

Figure S3. Molecular structure of the anion in compound **A**·py (left) with labelling scheme (Symmetry code: i = 1–x, –y, 1–z), and unit cell view of **A**·py (right). Atoms are shown as thermal ellipsoids with 50% probability. The two-colored octants indicate that the two atom types are not distinguishable by means of normal X-ray diffraction, yet the atom types were assigned in accordance with quantum chemical calculations of the most stable isomer.

Table S3. Selected interatomic distances (in Å) and bond angles (in degrees) of the experimental structure of the anion in A·py. Symmetry code: i = 1–x, –y, 1–z.

**Atoms**	**Distance (exp)**	**Atoms**	**Distance (exp)**
Au1–Sn1	2.7401(8)	Sn1–Sb2	2.8625(10)
Au1–Sn2	2.7304(9)	Sn2–Sb1	2.8743(11)
Sn1–Sn2	3.1533(10)	Sn2–Sb2	2.8610(11)
Sn1–Sb1	2.8494(13)	Sb1–Sb2	2.8385(11)
**Atoms**	**Angles (exp)**	**Atoms**	**Angles (exp)**
Sn2 – Au1 – Sn2^i	180.0	Sb2^i – Sn2^i – Sn1^i	56.59(2)
Sn2 – Au1 – Sn1^i	109.60(3)	Au1 – Sn1 – Sb1	103.34(3)
Sn2^i – Au1 – Sn1^i	70.40(3)	Au1 – Sn1 – Sn2	54.66(2)
Sn1 – Sb1 – Sn2	66.86(3)	Au1 – Sn1 – Sb2	103.42(3)
Sb2 – Sb1 – Sn2	60.10(3)	Sb1 – Sn1 – Sn2	56.95(3)
Sb2 – Sb1 – Sn1	60.43(3)	Sb1 – Sn1 – Sb2	59.60(3)
Au1 – Sn2^i – Sb1^i	102.93(4)	Sb2 – Sn1 – Sn2	56.55(2)
Au1 – Sn2^i – Sn1^i	54.95(2)	Sb1 – Sb2 – Sn2	60.57(3)
Au1 – Sn2^i – Sb2^i	103.71(2)	Sb1 – Sb2 – Sn1	59.97(3)
Sb1^i – Sn2^i – Sn1^i	56.19(3)	Sn2 – Sb2 – Sn1	66.86(3)
Sb2 – Sn2^i – Sb1^i	59.33(2)		

3 Energy-Dispersive X-Ray Spectroscopy (EDX)

EDX analysis of single crystals of compound **1** was carried out using an EDX-device Voyager 4.0 of Noran Instruments coupled with an electron microscope CamScan CS 4DV. Data acquisition was performed with an acceleration voltage of 15 kV and 100 s accumulation time. Results are summarized in Table S4. Note that the deviation of the K content is observed very frequently in EDX analyses of these very air-sensitive Zintl cluster compounds. The REM image of the investigated sample is shown along with the spectrum in Figure S4.

Table S4. EDX analysis of 1 (K, Au, Sb).

Element	Mass (%)	Atom (%)	Abs. error (1 sigma)	Element ratio calc
K-K	1.67	27.89	0.10	1.75
Au-L	19.15	63.64	0.85	4.00
Sb-L	1.57	8.47	0.10	0.53
Total	100.00	100.00		

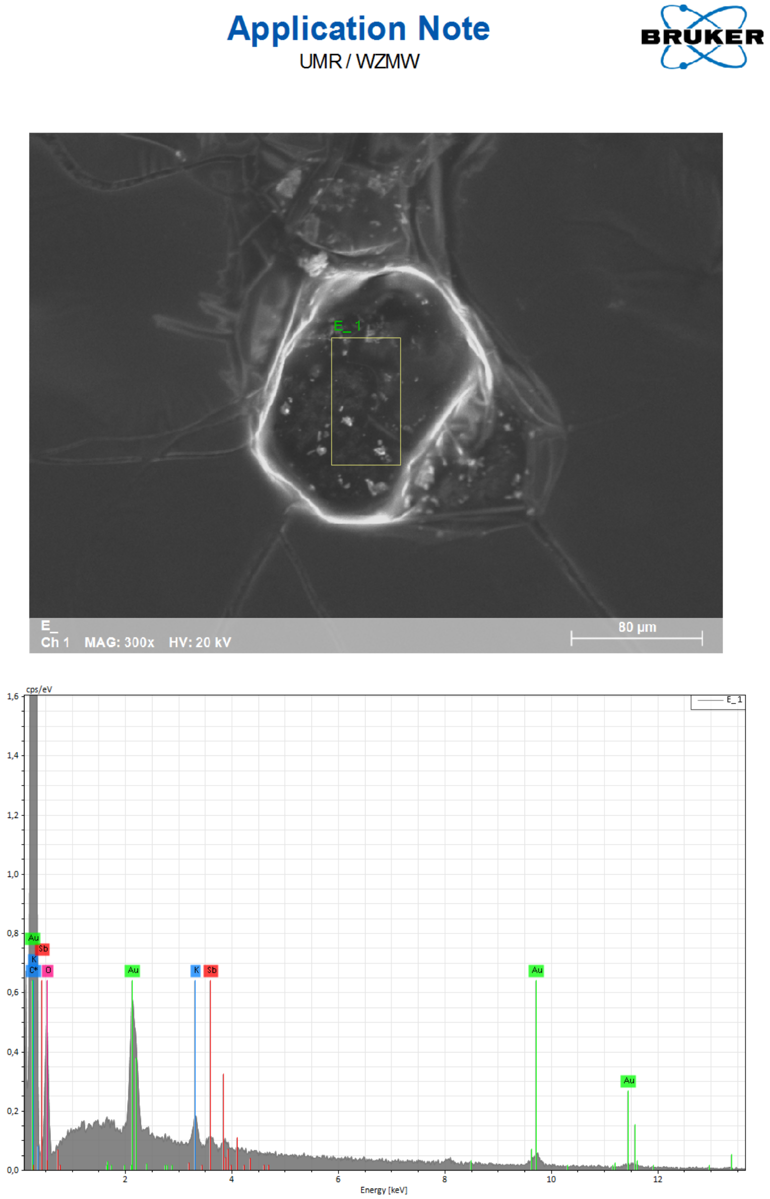

Figure S4. EDX analysis of **1**. REM image of the sample (top) and spectrum (bottom).

4 Electrospray Ionization Mass Spectrometry (ESI-MS)

4.1 Methods

All mass spectra were recorded with a Thermo Fischer Scientific Finnigan LTQ-FT spectrometer in negative ion mode. We prepared a fresh solution of single crystals of the compound **1** in freshly distilled DMF inside a glovebox. The solutions were injected into the spectrometer with gastight 250 µL Hamilton syringes by syringe pump infusion. All capillaries within the system were washed with dry DMF for 2 hours before and at least 10 minutes in between measurements to avoid decomposition reactions and consequent clogging.

4.2 ESI(−) Mass Spectrum of 1

The following ESI parameters were used: Spray Voltage: 3.6 kV, Capillary Temp: 290 °C, Capillary Voltage: −20, Tube lens Voltage: −121.75, Sheath Gas: 45, Sweep Gas: 0, Auxiliary Gas: 40. The overview mass spectrum in the range 150-2000 m/z is provided in Figure S5, assignable high-resolution mass peaks are shown in Figures S6-S10. Note that all species were detected as monoanions, which does not necessarily correspond to their original charge in solution.

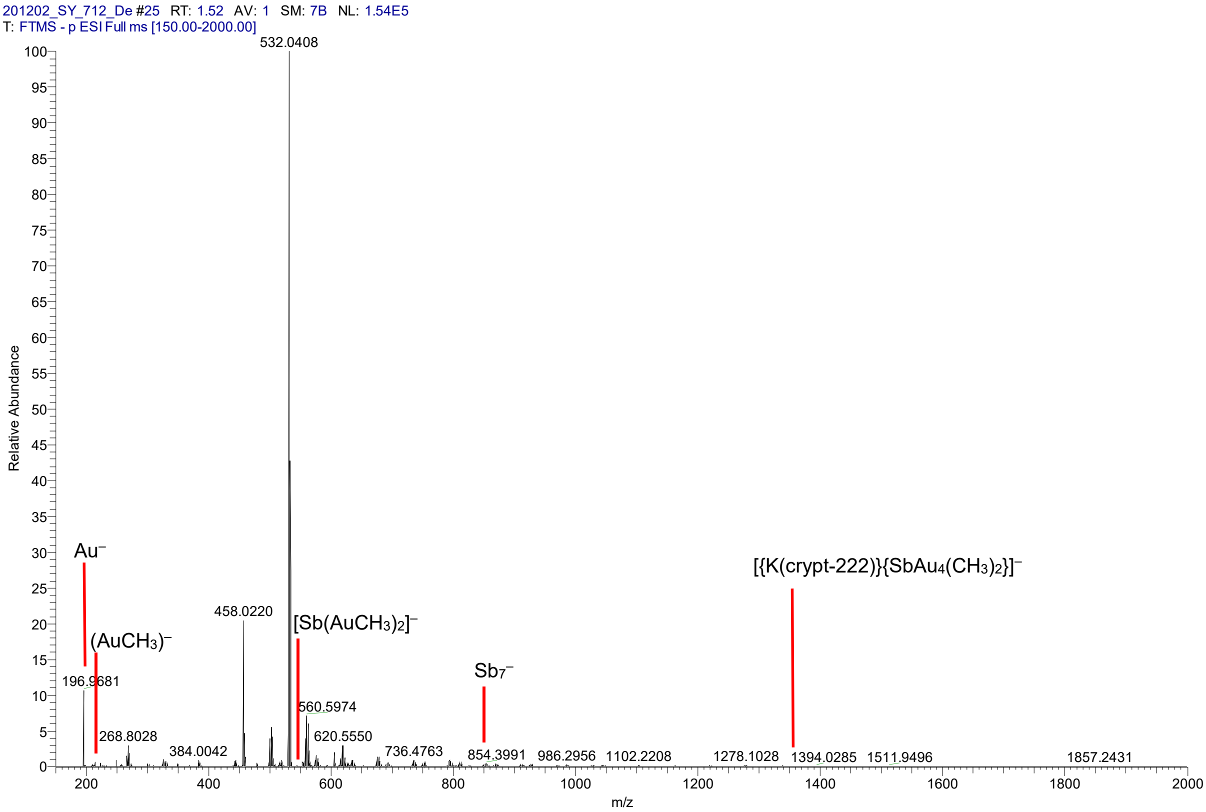

Figure S5. Overview ESI(−) mass spectrum recorded immediately upon injection of a fresh solution of **1** in DMF.

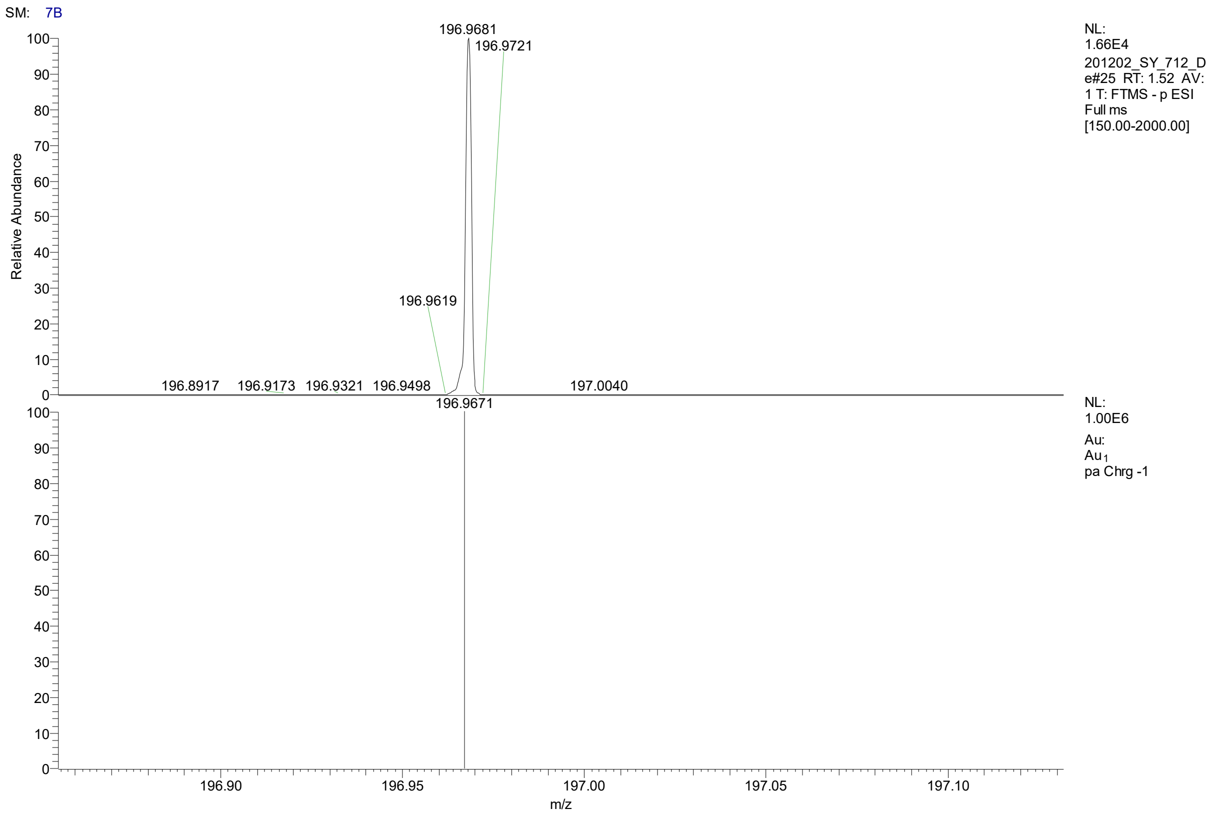

Figure S6. High-resolution ESI mass spectrum in negative ion mode of Au^{-}, recorded immediately upon injection of a fresh solution of **1** in DMF. Top: measured, bottom: simulated.

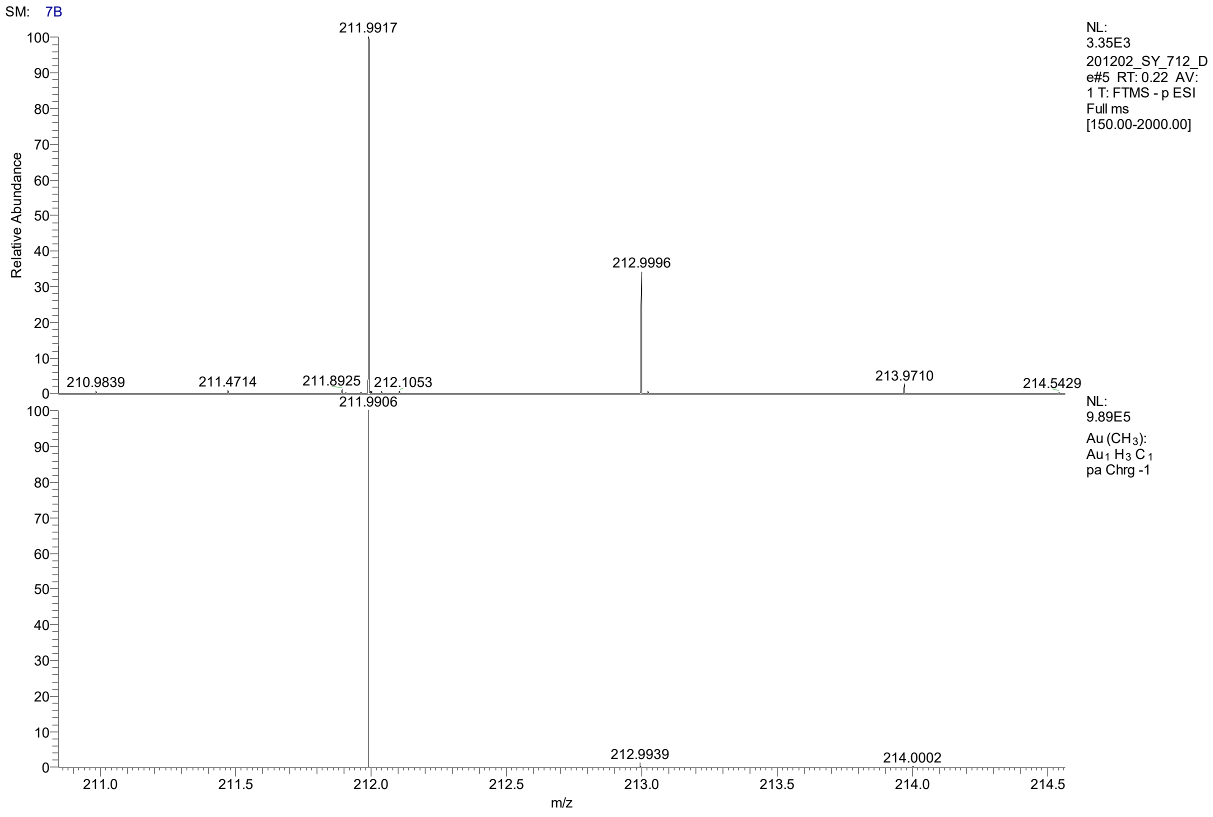

Figure S7. High-resolution ESI mass spectrum in negative ion mode of $(AuCH_3)^{-}$, recorded immediately upon injection of a fresh solution of **1** in DMF. Top: measured, bottom: simulated.

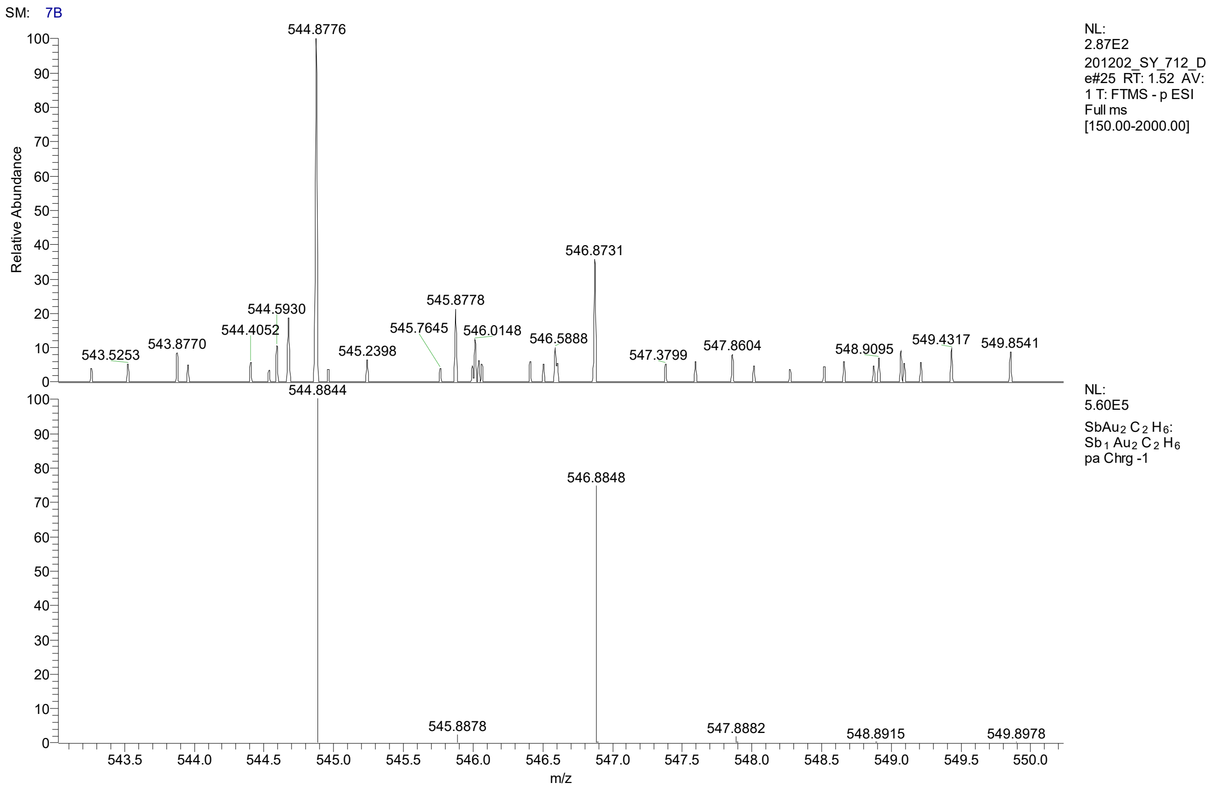

Figure S8. High-resolution ESI mass spectrum in negative ion mode of $[Sb(AuCH_3)_2]^-$, recorded immediately upon injection of a fresh solution of **1** in DMF. Top: measured, bottom: simulated.

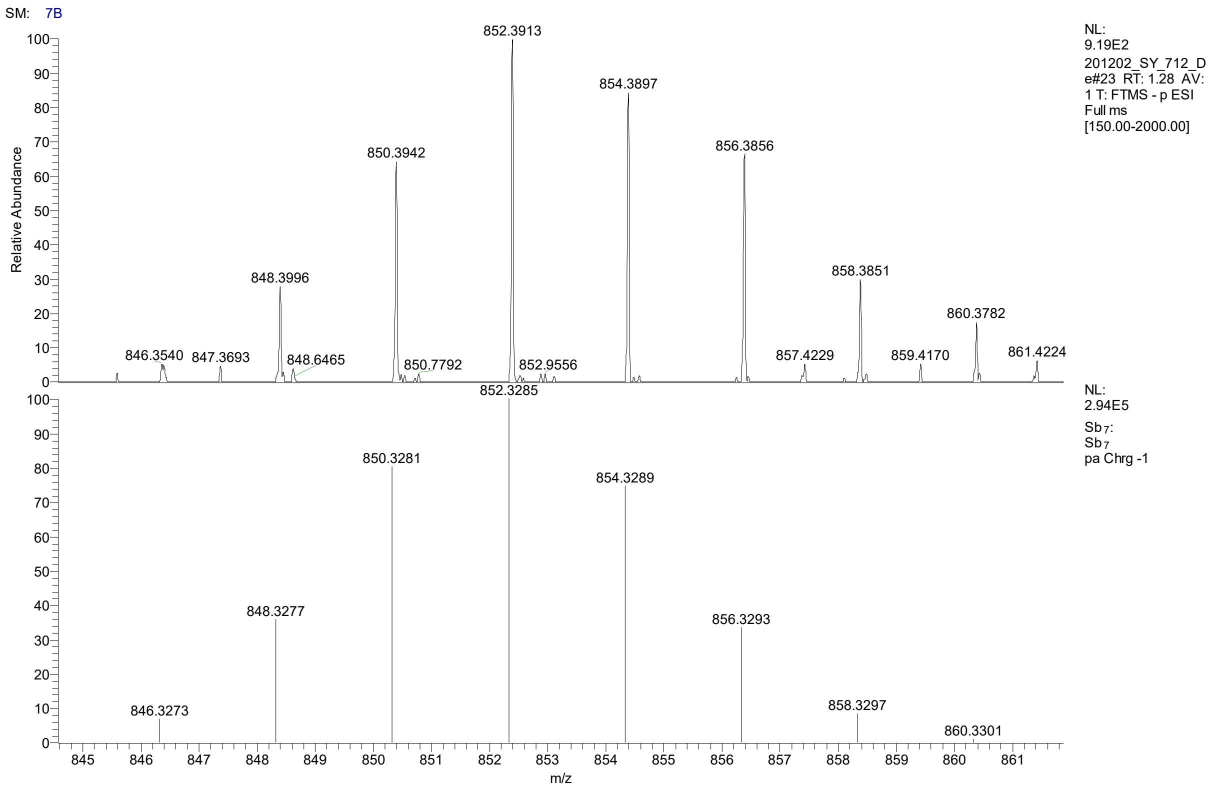

Figure S9. High-resolution ESI mass spectrum in negative ion mode of Sb_7^-, recorded immediately upon injection of a fresh solution of **1** in DMF. Top: measured, bottom: simulated.

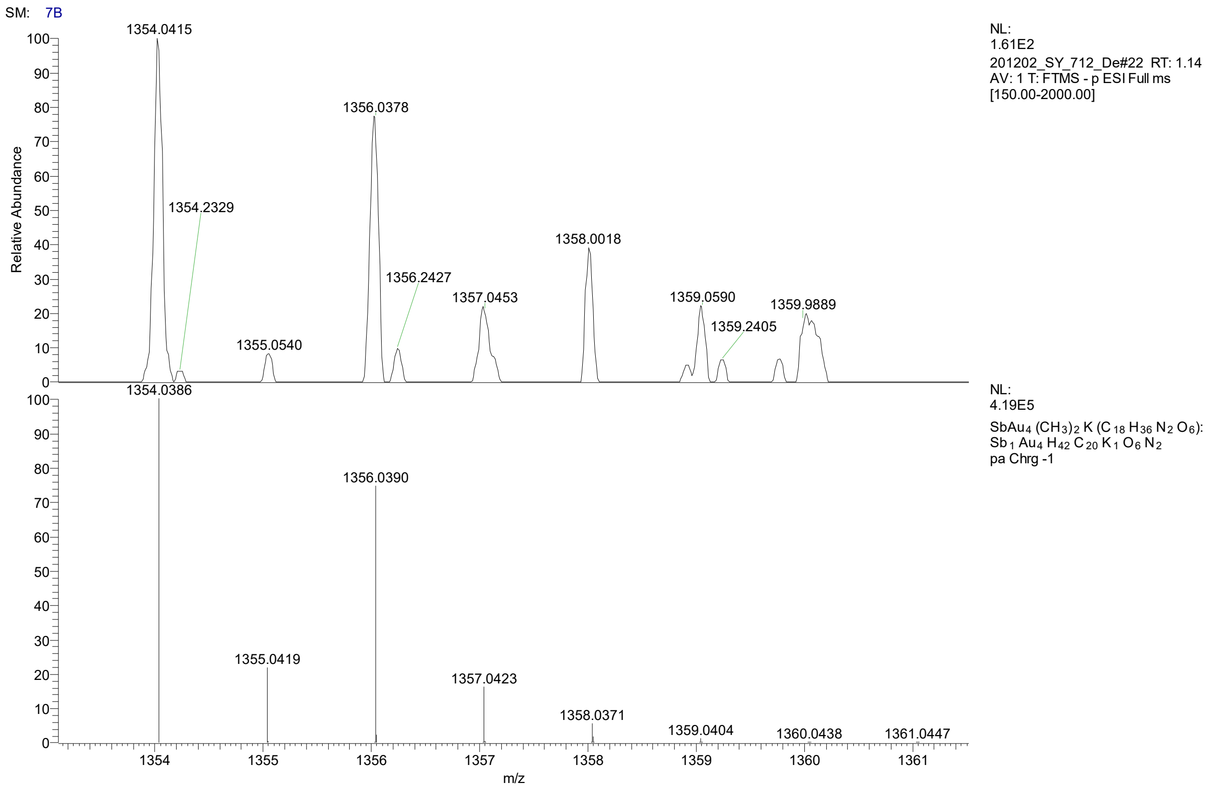

Figure S10. High-resolution ESI mass spectrum in negative ion mode of [{K(crypt-222)}{$SbAu_4(CH_3)_2$}]$^-$, recorded immediately upon injection of a fresh solution of **1** in DMF. Top: measured, bottom: simulated.

4.3 ESI(+) Mass Spectrum of 1

The following ESI parameters were used: Spray Voltage: 3.6 kV, Capillary Temp: 290 °C, Capillary Voltage: −20, Tube lens Voltage: −121.75, Sheath Gas: 45, Sweep Gas: 0, Auxiliary Gas: 40. The overview mass spectrum in the range 150-2000 m/z is provided in Figure S11, the high-resolution mass peaks of the monocation $[K(crypt\text{-}222)]^+$ at 415.2208 m/z is shown in Figure S12.

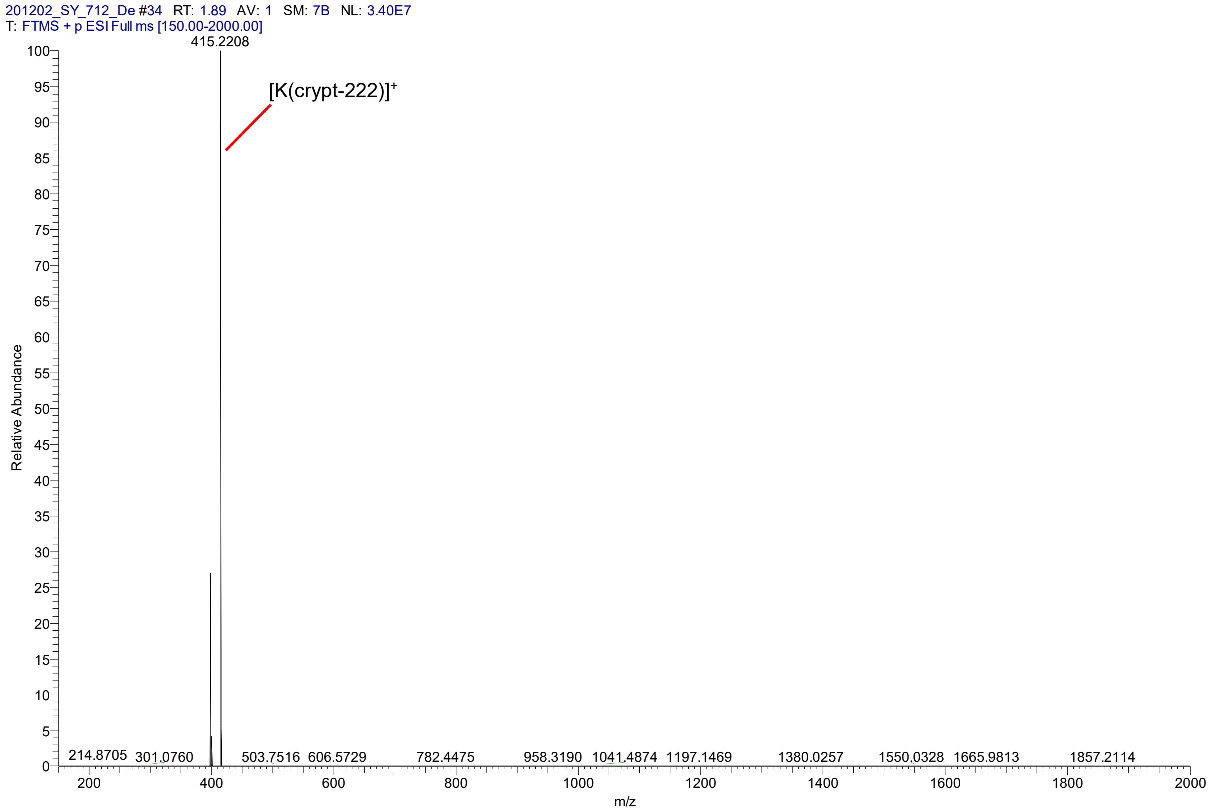

Figure S11. Overview ESI(+) mass spectrum recorded immediately upon injection of a fresh solution of **1** in DMF.

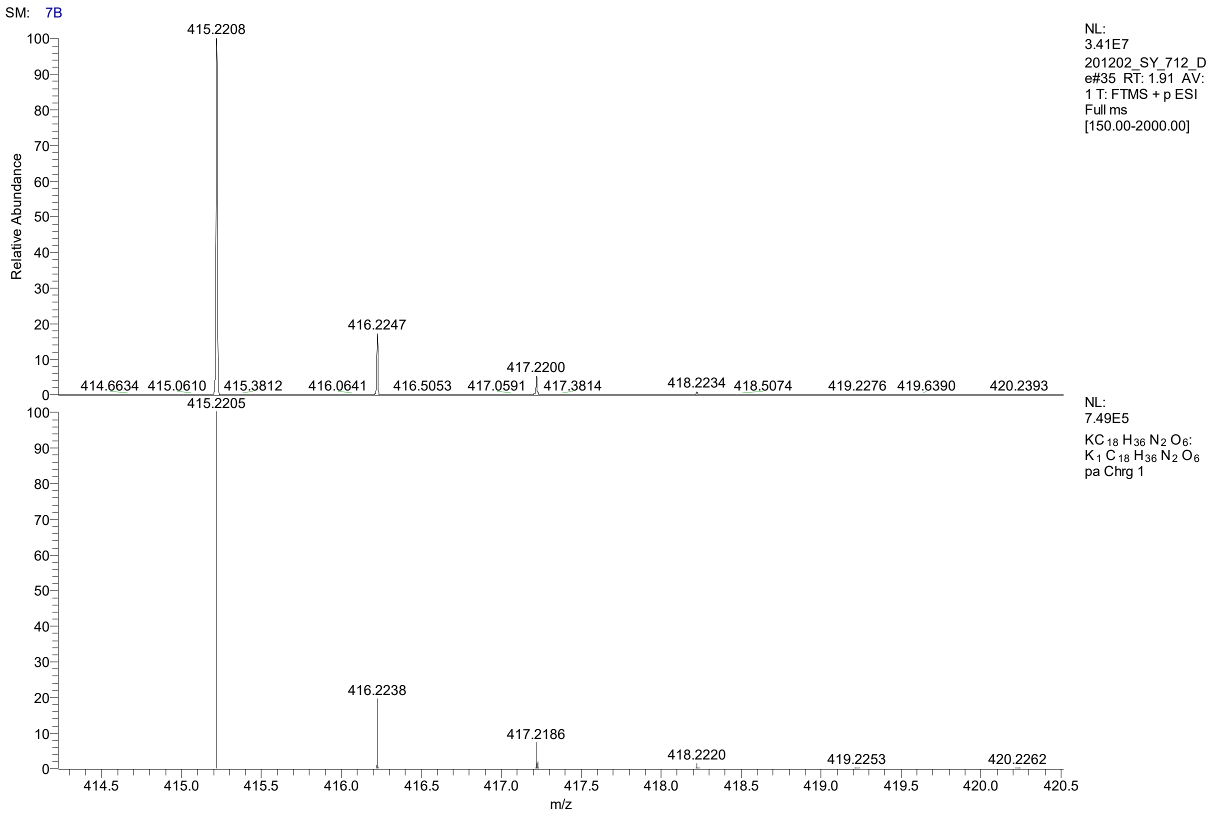

Figure S12. High-resolution ESI mass spectrum in positive ion mode of [K(crypt-222)]$^+$, recorded immediately upon injection of a fresh solution of **1** in DMF. Top: measured, bottom: simulated.

5 Characterization of Side-Products of Cluster Formation Reactions

5.1 EDX analysis of the Solid Residue upon Filtration of the Reaction Mixture to Form Compound 1

The EDX analysis was carried out using an EDX-device Voyager 4.0 of Noran Instruments coupled with an electron microscope CamScan CS 4DV. Data acquisition was performed with an acceleration voltage of 15 kV and 100 s accumulation time. Results are summarized in Table S5. The REM image of the investigated sample is shown along with the spectrum in Figure S13. There are considerable overlaps of K/Sn and Sn/Sb peaks, respectively, resulting in large abs. error. values. Thus, the element ratios of the residue was not calculated. However, the data clearly indicates the presence of potassium, tin, and antimony in the residue.

Table S5. EDX analysis of the solid residue upon filtration of the reaction mixture to form compound 1 (K, Au, Sb).

Element	Mass (%)	Atom (%)	Abs. error (1 sigma)
K-K	48.41	74.35	3.22
Sb-L	35.07	17.29	15.01
Sn-L	16.53	8.36	12.15
Total	100.00	100.00	

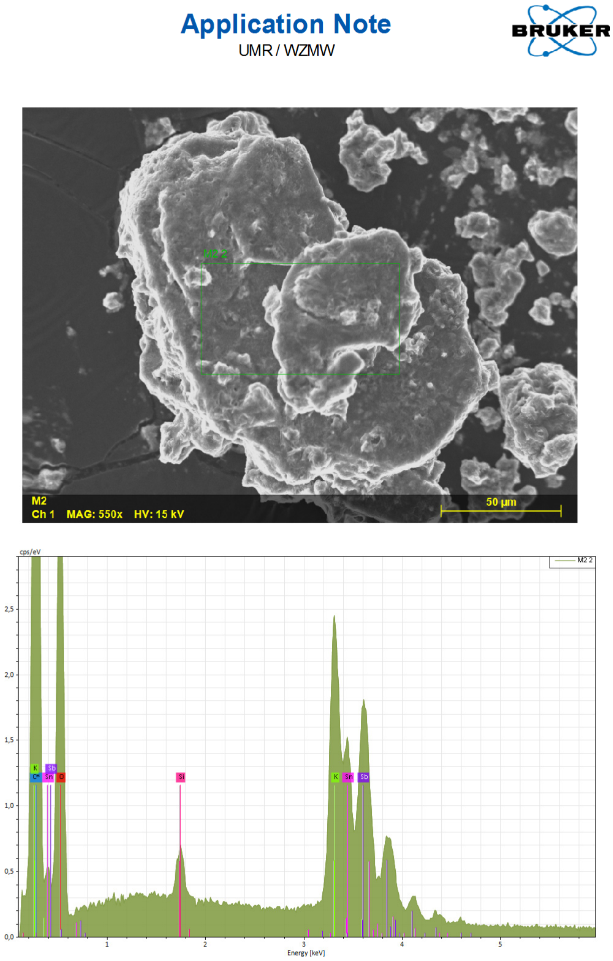

Figure S13. EDX analysis of the solid residue after filtration of the reaction mixture. REM image of the sample (top) and spectrum (bottom). The presence of Si in the sample is due to abrasion of glass from the glass-coated stirring bar and the glass tube while vigorously stirring the reaction mixture.

5.2 Powder X-Ray Diffraction (PXRD) of the Solid Residue upon Filtration of the Reaction Mixture to Form Compound 1

Powder X-ray diffraction (PXRD) data were collected on a Stoe StadiMP diffractometer system equipped with a Mythen 1 K silicon strip detector and Cu-K$_{\alpha}$-radiation (λ = 1.54056 Å). A sample of the solid residue upon filtration of the reaction mixture after 3h reaction time, and washing of the precipitate with py for 3 times, was filled into a glass capillary (0.3 mm diameter), which was sealed air-tightly with soft wax. The tube was then mounted onto the goniometer head using wax (horizontal setup) and rotated throughout the measurement. The diffraction pattern is shown in Figure S14. Figure S15 shows the diffraction pattern of the starting material [K(crypt-222)]$_2$(Sn$_2$Sb$_2$)·en for comparison. The diffraction pattern in Figure S14 shows the elements Sn and Sb to be present in crystalline form, while the starting material is not observed.

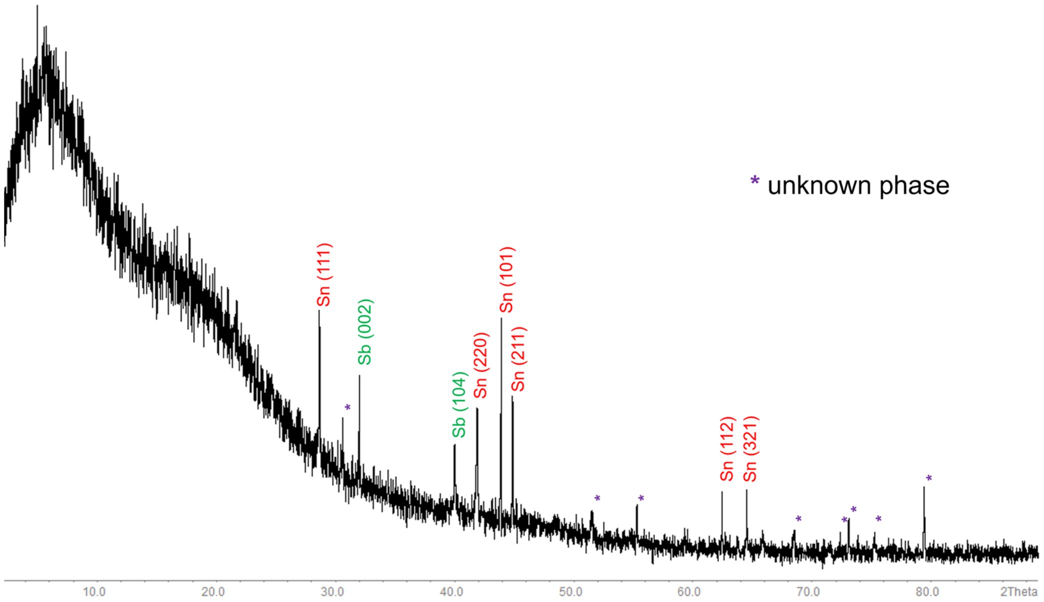

Figure S14. PXRD pattern of the solid residue upon filtration of the reaction mixture for the formation of compound **1**, after 3h reaction time, and washing of the precipitate with py for 3 times. Reflections that are indicative for the respective elements are assigned.

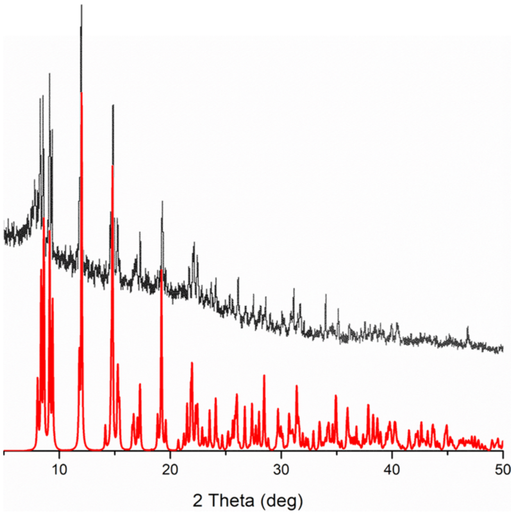

Figure S15. PXRD pattern of [K(crypt-222)]$_2$(Sn$_2$Sb$_2$)·en for comparison as measured (black line) and simulated from the single-crystal CIF deposited as CCDC 742876.

5.3 ^{31}P-NMR Analysis (en, py) of PPh_3, [$AuMePPh_3$], and the Reaction Mixtures of (Sn_2Sb_2)$^{2-}$ and [$AuMePPh_3$] to Form Compounds A (2:1 in en) or 1/B (1:2 in py)

^{31}P-NMR spectra of PPh_3, [$AuMePPh_3$] (in en and py), as well as reaction mixtures of (Sn_2Sb_2)$^{2-}$ and [$AuMePPh_3$] to form compounds A (2:1 in en, recorded after 3h reaction time) or **1**/**B** (1:2 in py, recorded after 3h reaction time) were recorded on a Bruker DPX AV 500 at room temperature. The raw data were examined by means of MestReNova 6.0. The spectra are given in Figures S16-S21. They indicate full conversion of [$AuMePPh_3$] under release of PPh_3 after 3h reaction time.

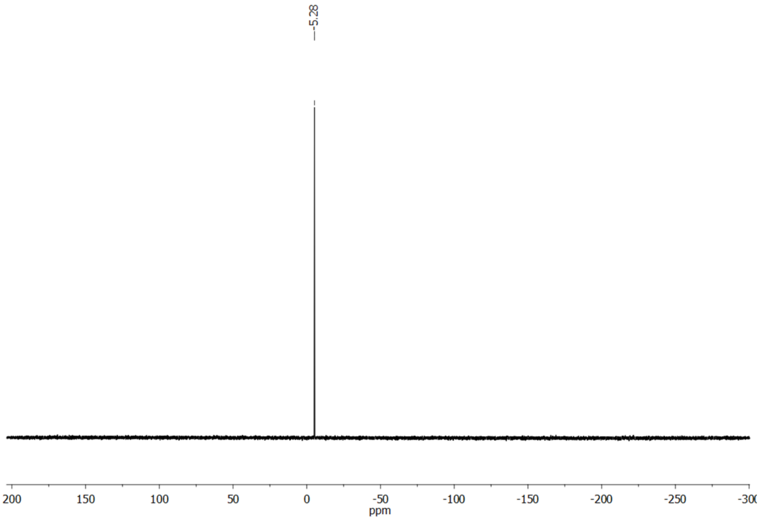

Figure S16. ^{31}P-NMR spectrum of PPh_3 in en.

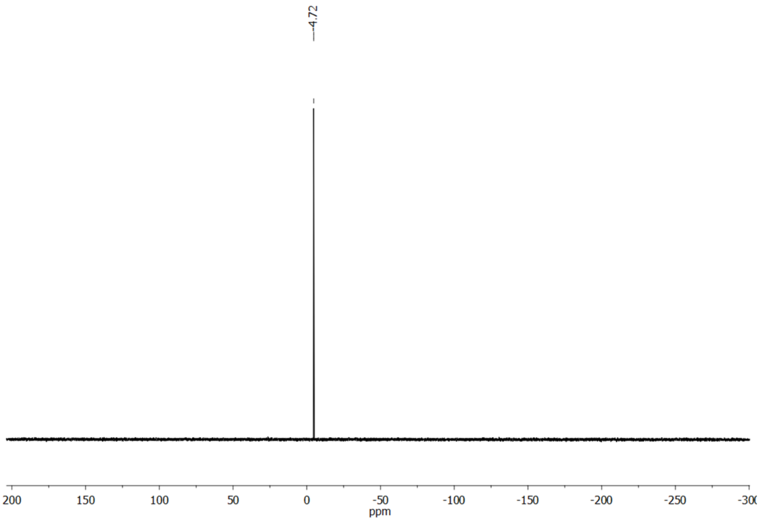

Figure S17. ^{31}P NMR spectrum of PPh_3 in py.

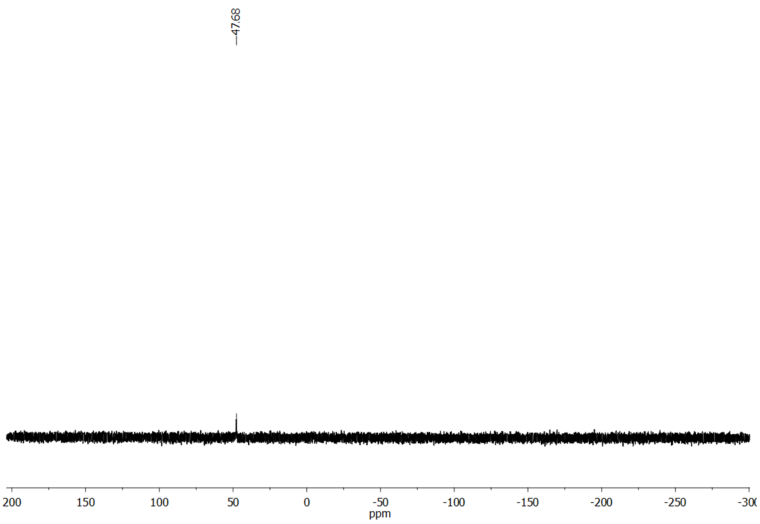

Figure S18. ^{31}P-NMR spectrum of [$AuMePPh_3$] in en. The low peak intensity is due to the low solubility of [$AuMePPh_3$] in en at room temperature.

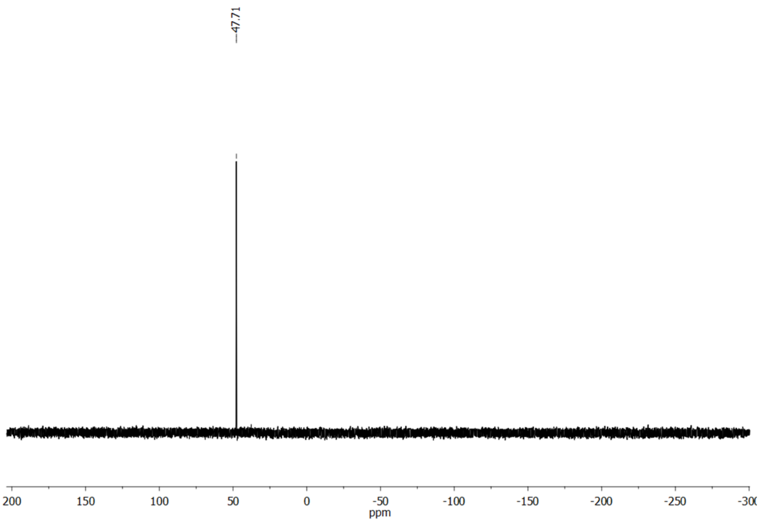

Figure S19. ^{31}P-NMR spectrum of [$AuMePPh_3$] in py.

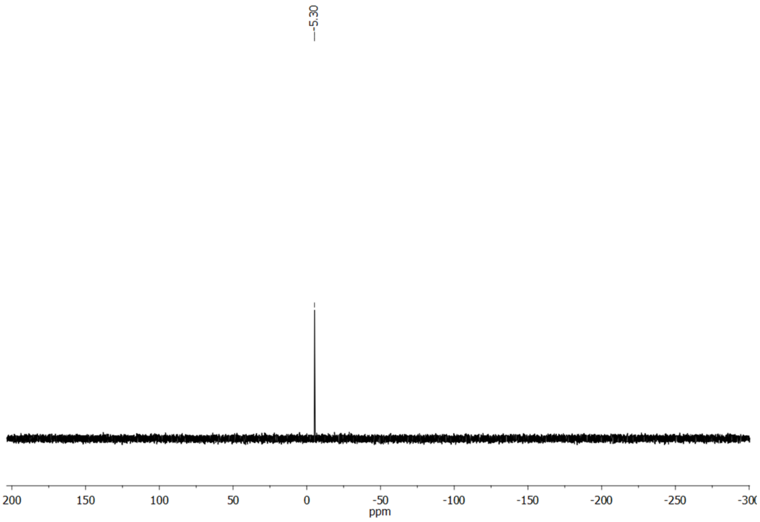

Figure S20. ^{31}P-NMR spectrum of a reaction solution of [K(crypt-222)]$_2$(Sn_2Sb_2)·en and [$AuMePPh_3$] in a 2:1 molar ratio in en, recorded after 3h reaction time.

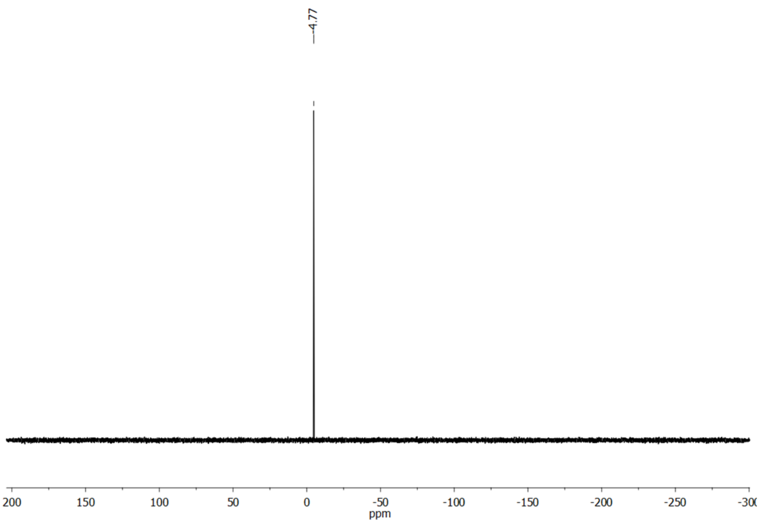

Figure S21. ^{31}P-NMR spectrum of a reaction solution of $[K(crypt\text{-}222)]_2(Sn_2Sb_2)\cdot en$ and $[AuMePPh_3]$ in a 1:2 molar ratio in py, recorded after 3h reaction time.

5.4 Electrospray Ionization Mass Spectrometry (ESI-MS) of the Reaction Solution to Form Compounds 1/B (1:2 in py)

The mass spectra was recorded with a Thermo Fischer Scientific Finnigan LTQ-FT spectrometer in negative and positive ion mode. The following ESI parameters were used for ESI(–/+): Spray Voltage: 3.6 kV, Capillary Temp: 290 °C, Capillary Voltage: −20, Tube lens Voltage: −121.75, Sheath Gas: 45, Sweep Gas: 0, Auxiliary Gas: 40.

We prepared a fresh solution of $[K(crypt\text{-}222)]_2(Sn_2Sb_2)\cdot en$ and $[AuMePPh_3]$ in a 1:2 ratio in py. After 3h reaction time, the solution was diluted with freshly distilled DMF inside a glovebox. The solution was injected into the spectrometer with a gastight 250 µL Hamilton syringe by syringe pump infusion. All capillaries within the system were washed with dry DMF for 2 hours before and at least 10 minutes in between measurements to avoid decomposition reactions and consequent clogging. The overview mass spectra in the range 50-500 m/z are provided in Figure S22 for ESI(–) and in Figure S26 for ESI(+), assignable high-resolution mass peaks are shown in Figures S23-S25 and S27, respectively. While the largest peaks (223.0283 m/z and 239.0597 m/z) are artefacts from the instrument, the following species could be assigned in the context of the reaction: Au^- (196,9675 m/z), $(HAuCH_3)^-$ (212.9986 m/z), and $[Au(CH_3)_2]^-$ (227.0143 m/z).

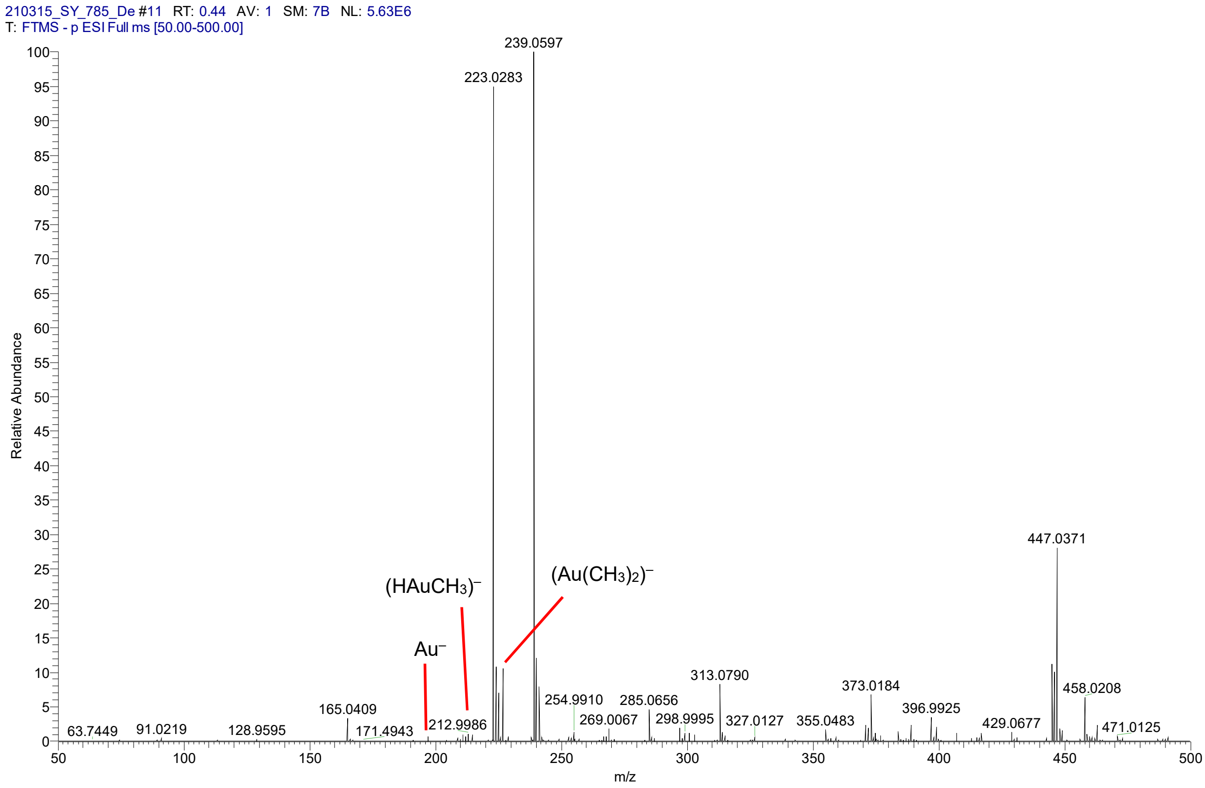

Figure S22. Overview ESI mass spectrum in negative ion mode, ESI(−), recorded immediately upon injection of a solution of $[K(crypt\text{-}222)]_2(Sn_2Sb_2)\cdot en$ and $[AuMePPh_3]$ in a 1:2 ratio in py, diluted with freshly distilled DMF inside a glovebox, after 3h reaction time.

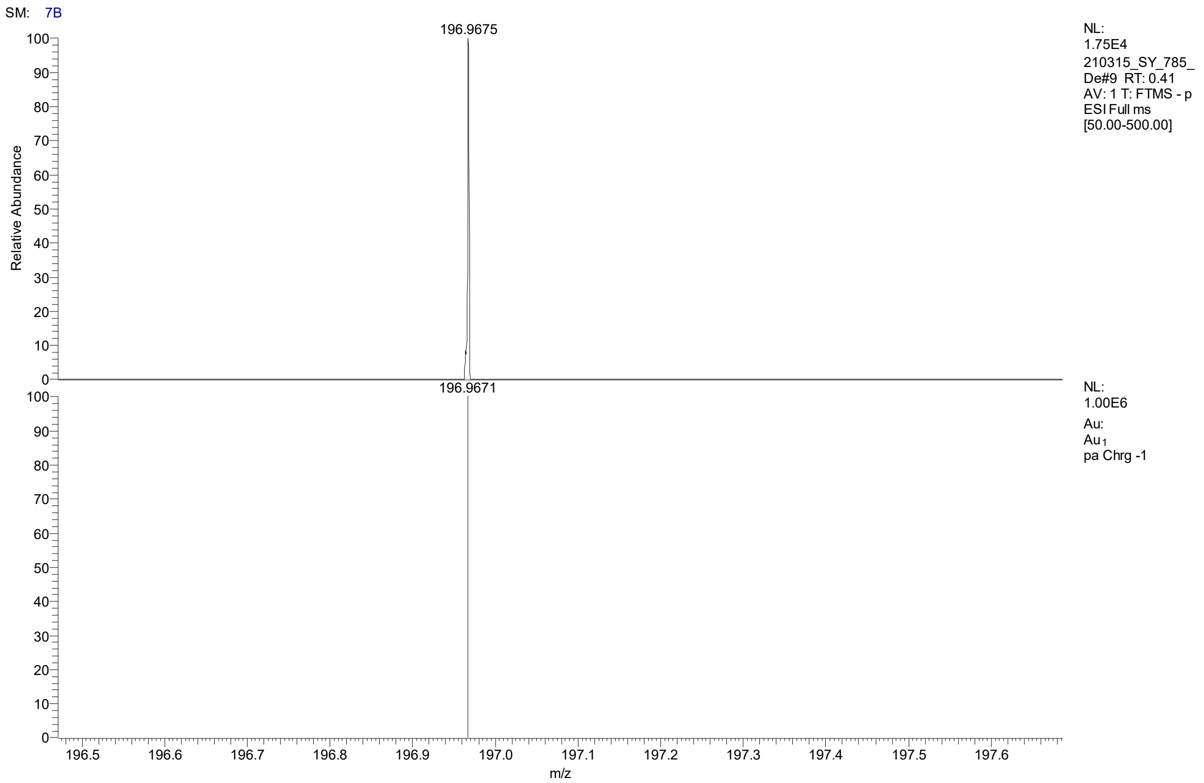

Figure S23. High-resolution ESI mass spectrum in negative ion mode of Au^{-}, recorded immediately upon injection of a solution of $[K(crypt\text{-}222)]_2(Sn_2Sb_2)\cdot en$ and $[AuMePPh_3]$ in a 1:2 ratio in py, diluted with freshly distilled DMF inside a glovebox, after 3h reaction time. Top: measured, bottom: simulated.

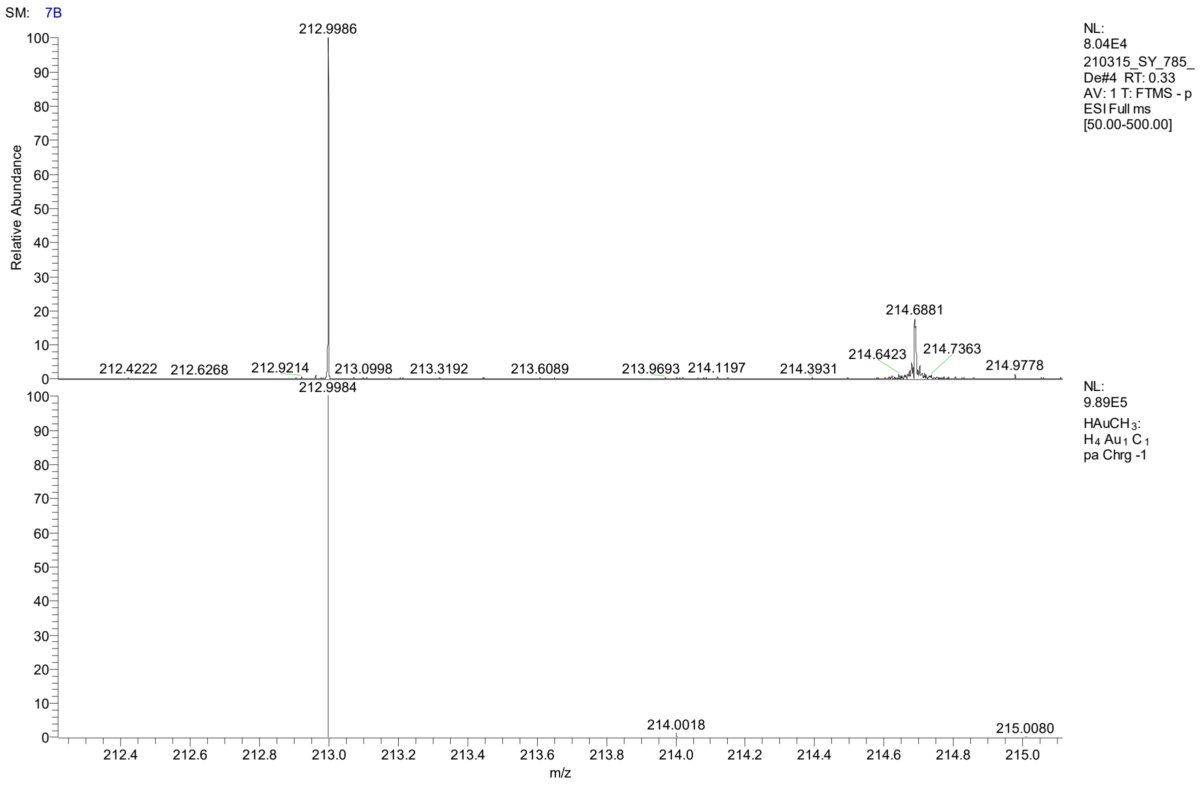

Figure S24. High-resolution ESI mass spectrum in negative ion mode of $(HAuCH_3)^{-}$, recorded immediately upon injection of a solution of $[K(crypt\text{-}222)]_2(Sn_2Sb_2)\cdot en$ and $[AuMePPh_3]$ in a 1:2 ratio in py, diluted with freshly distilled DMF inside a glovebox, after 3h reaction time. Top: measured, bottom: simulated.

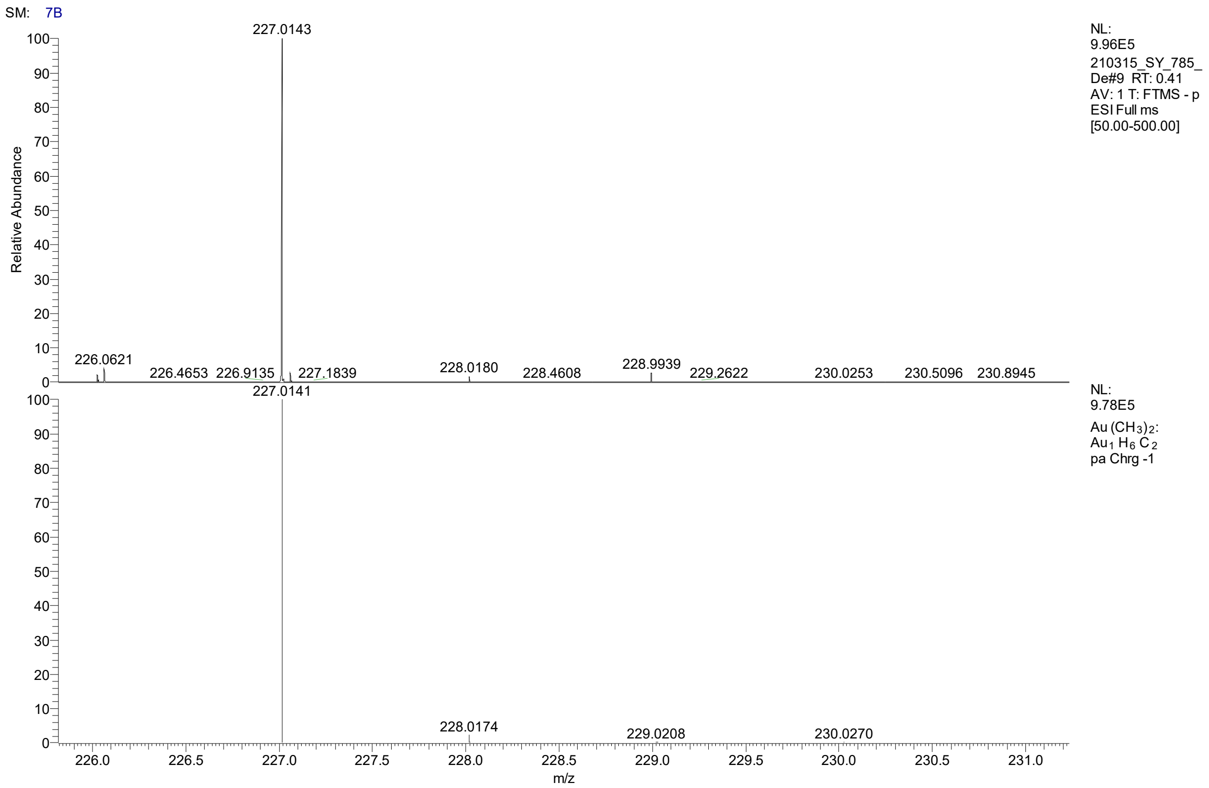

Figure S25. High-resolution ESI mass spectrum in negative ion mode of $[Au(CH_3)_2]^-$, recorded immediately upon injection of a solution of $[K(crypt\text{-}222)]_2(Sn_2Sb_2)\cdot en$ and $[AuMePPh_3]$ in a 1:2 ratio in py, diluted with freshly distilled DMF inside a glovebox, after 3h reaction time. Top: measured, bottom: simulated.

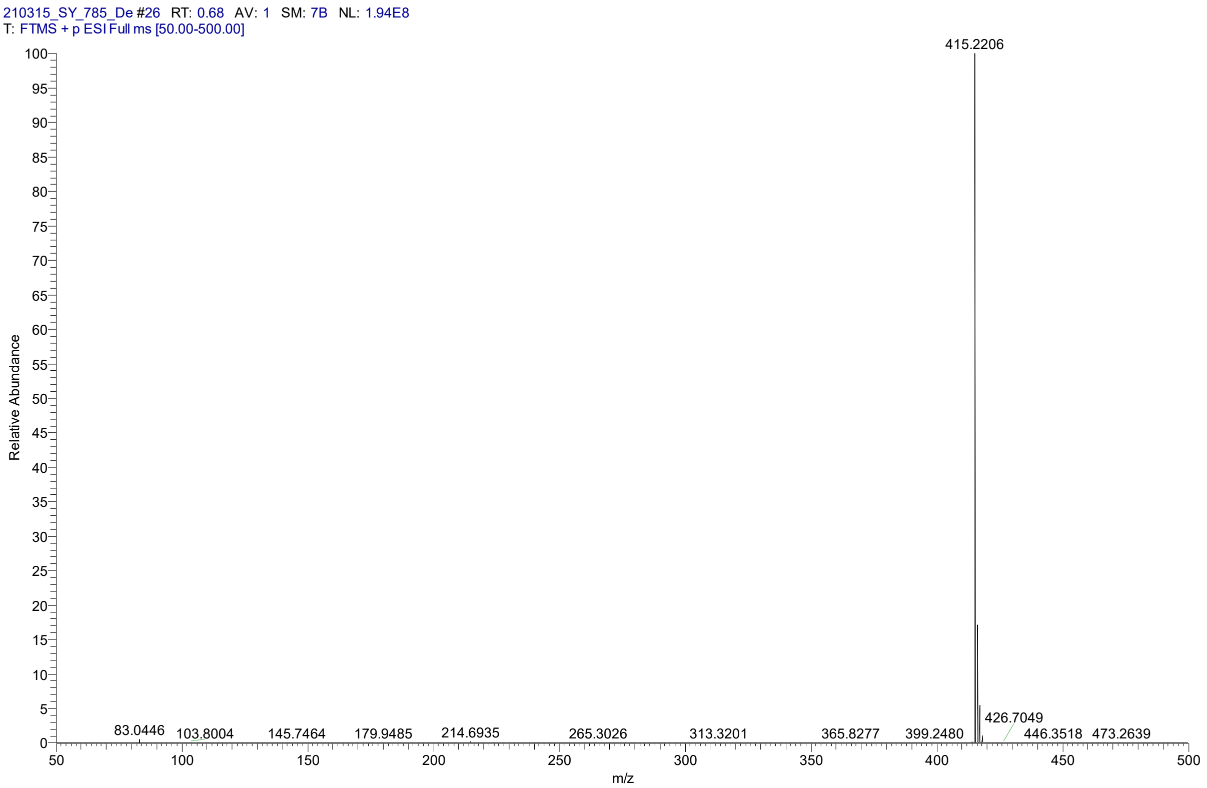

Figure S26 Overview ESI mass spectrum in positive ion mode, ESI(+), recorded immediately upon injection of a solution of [K(crypt-222)]$_2$(Sn$_2$Sb$_2$)·en and [AuMePPh$_3$] in a 1:2 ratio in py, diluted with freshly distilled DMF inside a glovebox, after 3h reaction time.

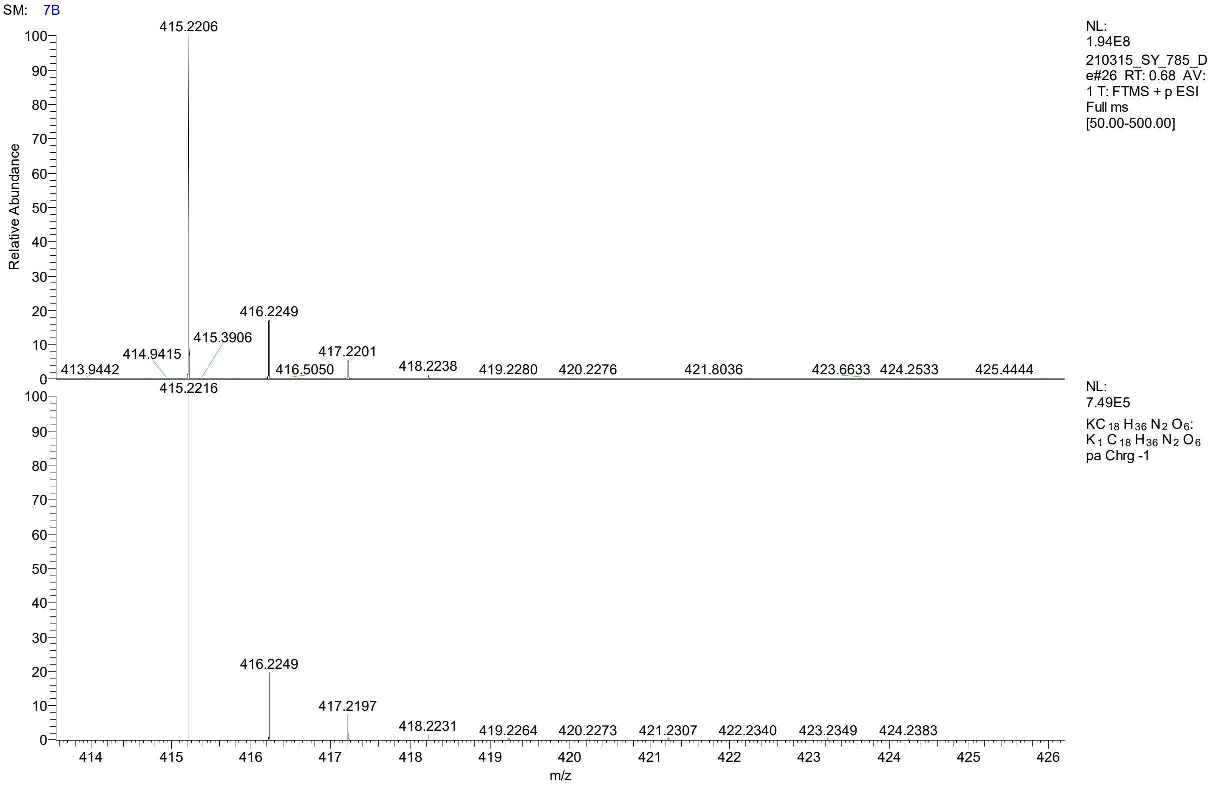

Figure S27. High-resolution ESI mass spectrum in positive ion mode of [K(crypt-222)]$^+$, recorded immediately upon injection of a solution of [K(crypt-222)]$_2$(Sn$_2$Sb$_2$)·en and [AuMePPh$_3$] in a 1:2 ratio in py, diluted with freshly distilled DMF inside a glovebox, after 3h reaction time. Top: measured, bottom: simulated.

5.5 CH_4 Detection by Gas Chromatography-Mass Spectrometry (GC-MS) Analysis of the Gas-Phase above a Reaction Solution to Form Compounds 1/B (1:2 in py)

To validate the CH_4 release that was proposed by quantum chemical studies for a reaction mixture of $(Sn_2Sb_2)^{2-}$ and [AuMePPh$_3$] to form compounds **1**/**B** (1:2 in py, see Scheme 2 in the main document and equation D1 in Table S18), gas chromatography-mass spectrometry (GC-MS) analyses were performed on a 5973N/6890N Single-Quadrupol GC-MS-System from Agilent. The gas above py solutions comprising either only [K(crypt-222)]$_2$(Sn$_2$Sb$_2$)·en, or [AuMePPh$_3$], or a 1:2 mixture of both (after 5 min or 1.5h reaction time, respectively), was loaded into a syringe upon piercing the rubber septum (that tightly closed the respective Schlenk tube) with a hollow needle, and immediately injected into the instrument. The results are illustrated in Figures S28 – S31. They prove the formation of CH_4 already after 5 min reaction time, in contrast to no CH_4 in solutions of the separated reactants.

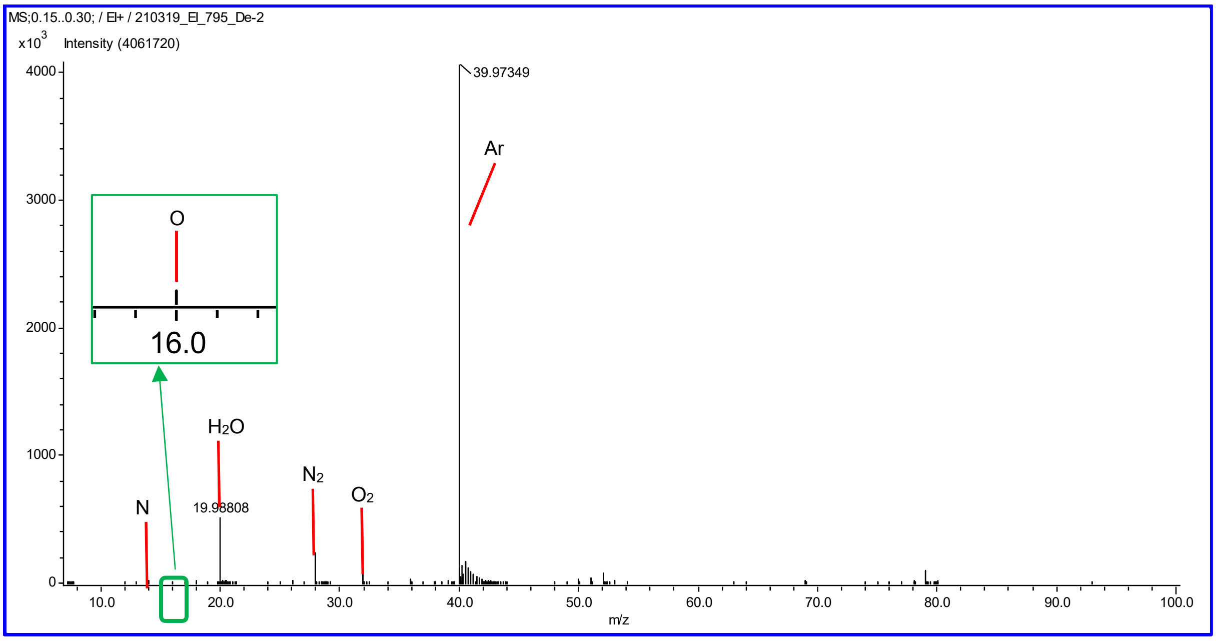

Figure S28. Py solution of [K(crypt-222)]$_2$(Sn$_2$Sb$_2$)·en (stirred for 5 min prior to injection).

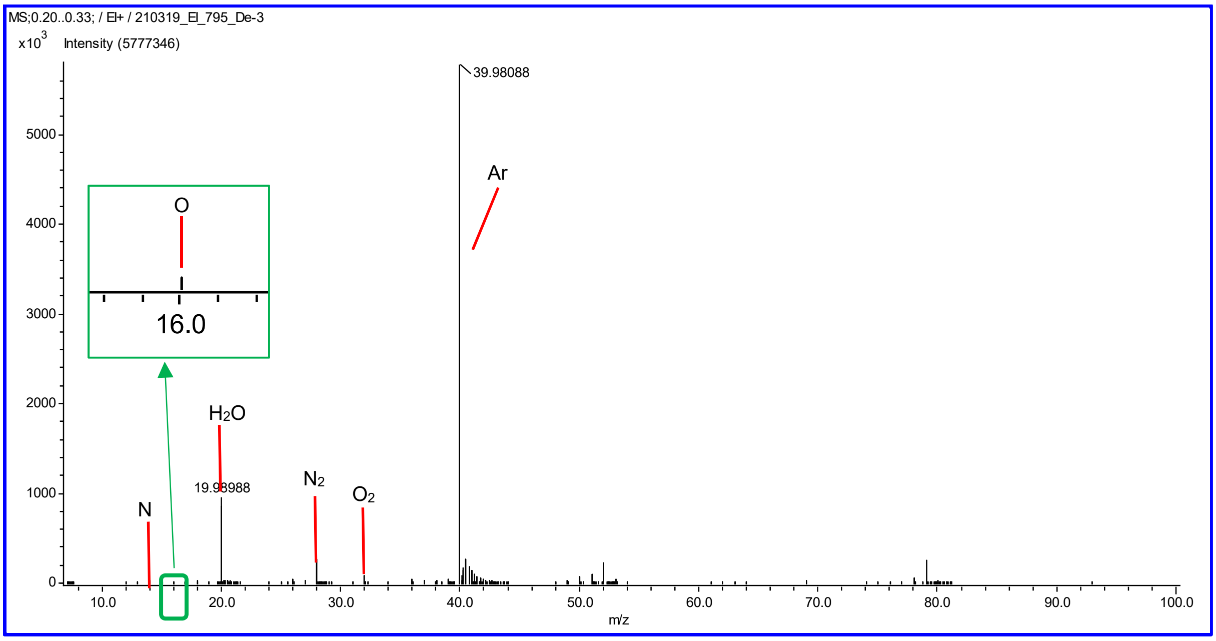

Figure S29. Py solution of [AuMePPh$_3$] (stirred for 5 min prior to injection).

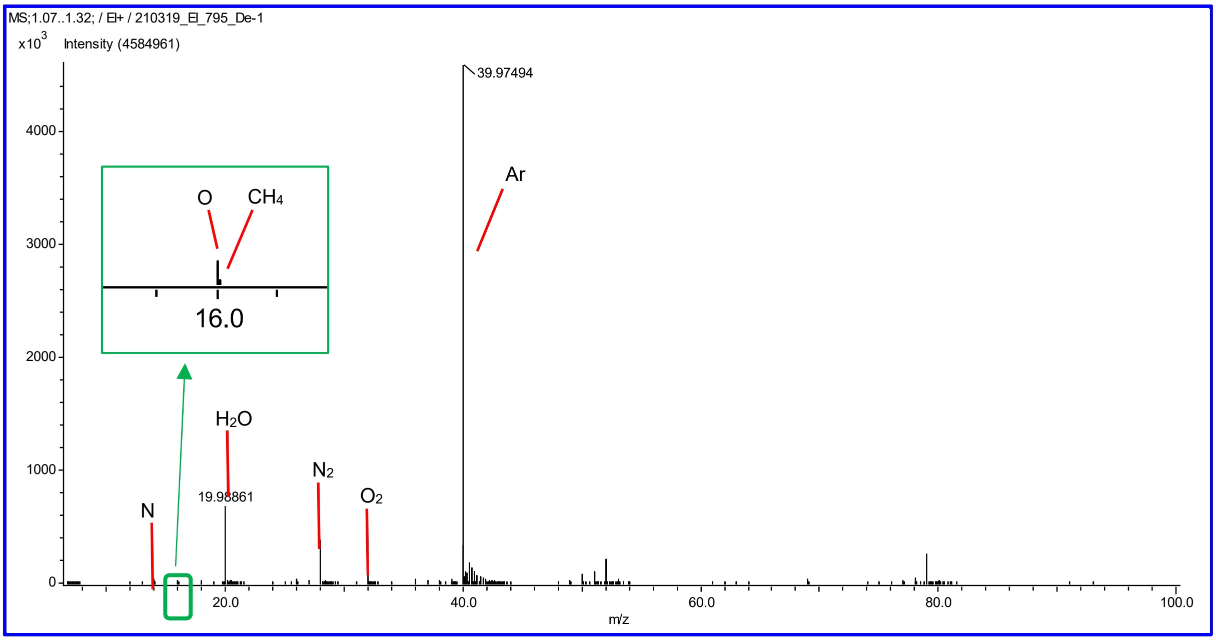

Figure S30. Reaction solution of [K(crypt-222)]2(Sn2Sb2)·en and [AuMePPh3] in a 1:2 molar ratio in py (stirred for 5 min prior to injection).

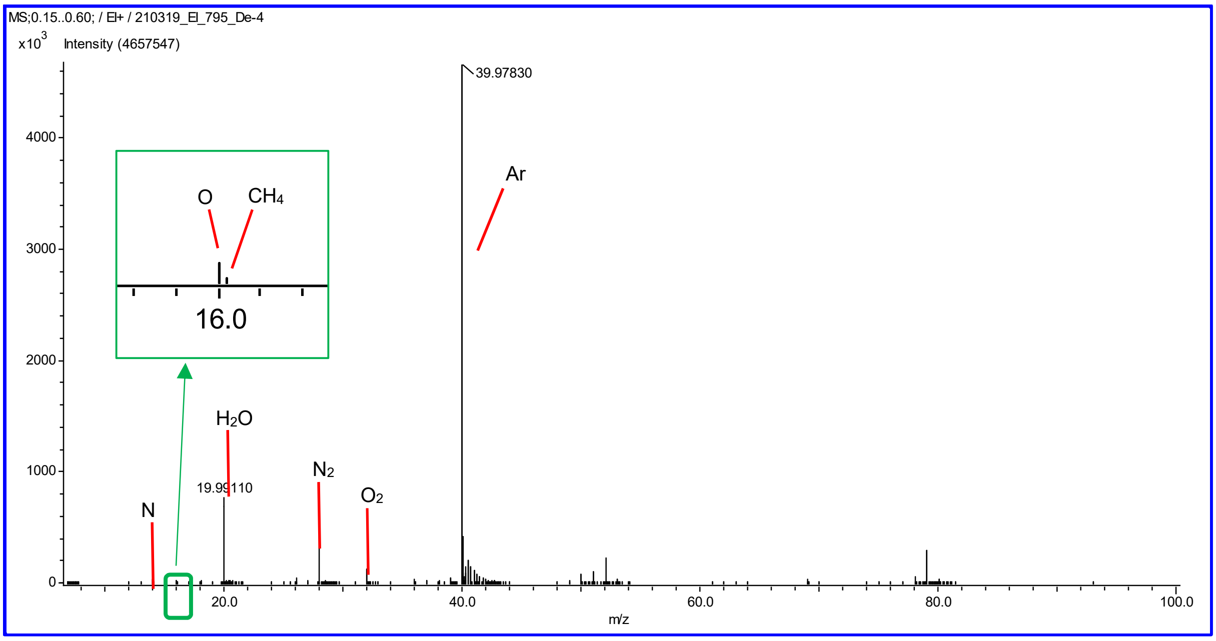

Figure S31. Reaction solutions of [K(crypt-222)]2(Sn2Sb2)·en and [AuMePPh3] in a 1:2 molar ratio in py (stirred for 1.5 h prior to injection).

5.6 Hydrogen Detection in the Reaction Solution to Form Compounds A (2:1 in en) or 1/B (1:2 in py)

To validate the H_2 release that was proposed by quantum chemical studies for reaction mixtures of $(Sn_2Sb_2)^{2-}$ and [AuMePPh$_3$] to form compound **A** (2:1 in en, see Scheme 2 in the main document and equation B3 in Table S13) or compounds **1**/**B** (1:2 in py, see Scheme 2 in the main document and equation D1 in Table S13) were prepared prior to the test.

After 3h reaction time, the release of hydrogen was probed according to a previously reported method,[7] yet by using metallic palladium powder instead of palladium plates for the absorption of H_2.

(1) Preparation of the indicator: Molybdenum trioxide (1 g) was dissolved in 5 mL of NaOH solution (20%). 7 mL of an HCl solution (20%) was added, and the mixture was diluted by water to adopt a total volume of 200 mL.

(2) Palladium powder (0.2 g) was placed in a 15 mL Schlenk flask, which was connected to a bubbler. The metal powder was heated to 200 degree for 15 minutes under dynamic vacuum and washed with argon two times.

The atmosphere over the reaction solution was transferred by applying slight vacuum to the flask containing palladium. The connecters were closed, whereupon the flask was heated up to 200 degree for ten minutes. The H_2-loaded palladium powder was poured into the indicator solution.

As shown in Figures S32 and S33, the resulting colorless solution for the test of the reaction to form compound **A** indicates no H_2 release, while the light blue color observed during the corresponding analysis of the reaction to form compounds **1**/**B** indicates H_2 release.

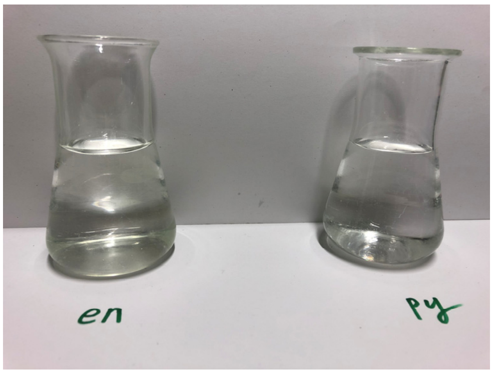

Figure S32. As-prepared indicator solutions.

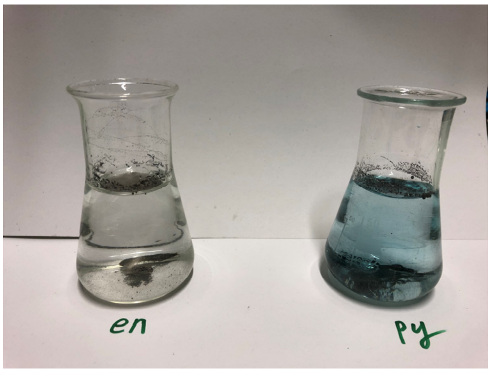

Figure S33. 0.5 h after pouring (H_2-loaded) palladium powder into the indicator solutions to probe the absence or presence of H_2, according the reactions given in equations (2b) and (4a) in Scheme 2 in the main document (left hand side and right hand side of the image, respectively).

6 Quantum Chemical Investigations

6.1 Investigations of the $[Sb(AuMe)_4]^{3-}$ anion in 1

Density functional theory (DFT) calculations were carried out to simultaneously optimize the geometric and electronic structure of the $[Sb(AuMe)_4]^{3-}$ anion in compound **1**. The calculations were done with the program system TURBOMOLE,[8] using the functional TPSS[9] and basis sets dhf-TZVP with corresponding auxiliary bases.[10] Effective core potentials were applied to model the Bi atoms.[11] To compensate for the negative charge of the molecule, COSMO[12] was employed using standard settings and an infinite dielectric constant. Force constants and the vibrational spectrum were calculated by application of the NumForce program. The absence of any imaginary frequencies proves the structure to be a local minimum.

The optimized structure of the calculated anion are shown in Figure S34. Selected MOs are provided in Figure S35. Localized molecular orbitals (LMOs) of the anion in **1** were calculated using the method of Boys and Foster.[13] Selected LMOs are provided in Figure S36. MO and LMO plots were created with gOpenMol.[14] The calculated vibrational spectrum is shown in Figure S37.

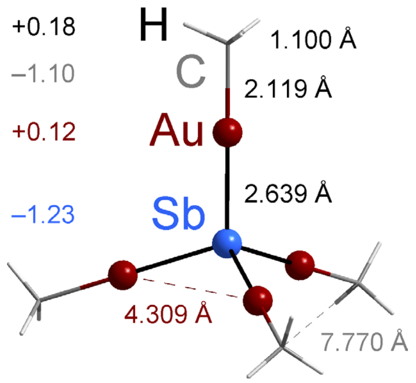

Figure S34. Calculated structure of the anion $[Sb(AuMe)_4]^{3-}$ optimized at the TPSS/dhf-TZVP level of theory in T_d symmetry, indicating structural parameters (at the bonds) and natural charges of the atoms (to the left; the color code is that of the atoms).

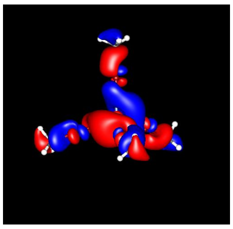

$13t_2$ (HOMO)
$\varepsilon = -3.973$ eV

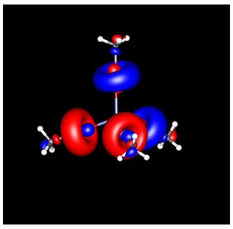

$12t_2$
$\varepsilon = -5.279$ eV

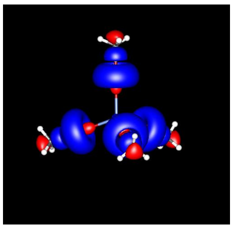

$8a_1$
$\varepsilon = -5.419$ eV

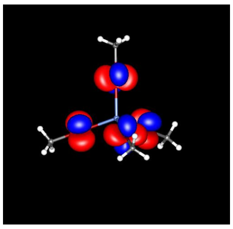

5e
$\varepsilon = -5.810$ eV

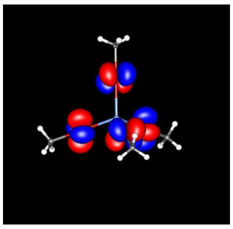

$4t_1$
$\varepsilon = -5.810$ eV

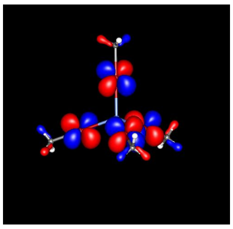

$3t_1$
$\varepsilon = -5.846$ eV

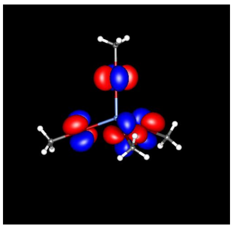

$11t_2$
$\varepsilon = -5.881$ eV

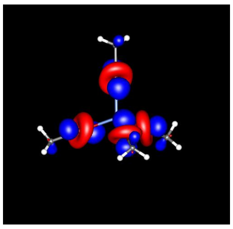

4e
$\varepsilon = -6.040$ eV

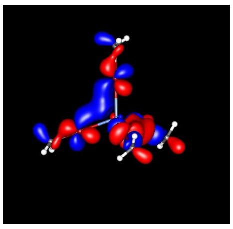

$10t_2$
$\varepsilon = -6.284$ eV

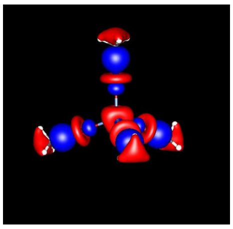

$7a_1$
$\varepsilon = -6.962$ eV

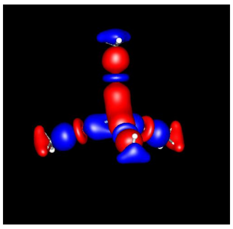

$9t_2$
$\varepsilon = -7.979$ eV

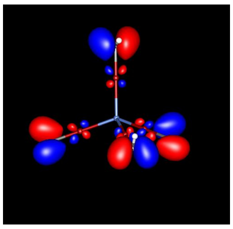

$2t_1$
$\varepsilon = -8.924$ eV

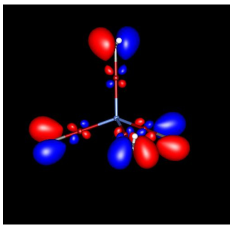

3e
$\varepsilon = -8.927$ eV

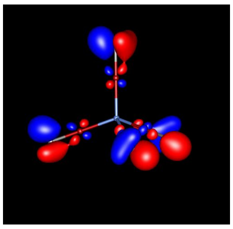

$8t_2$
$\varepsilon = -8.931$ eV

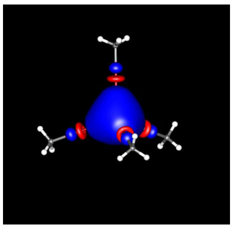

$6a_1$
$\varepsilon = -12.029$ eV

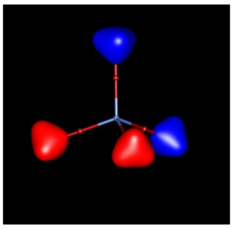

$7t_2$
$\varepsilon = -16.057$ eV

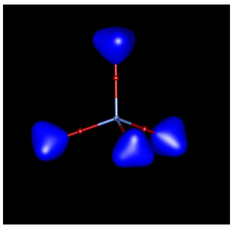

$5a_1$
$\varepsilon = -16.063$ eV

Figure S35. Plots of valence molecular orbitals (MOs) of the $[Sb(AuMe)_4]^{3-}$ anion in compound **1** (along with their respective energy eigenvalues). For doubly or triply degenerate MOs. only one representation is shown each. MOs $13t_2$ (HOMO), $9t_2$, $8a_1$, and $7a_1$ show major contributions to Sb–Au or Au–C interactions, while MOs $10t_2$ and $6a_1$ contribute to a minor extent to bonding only. MO $2t_1$, 3e, $8t_2$, $7t_2$, and $5a_1$ represent C–H bonds. All other MOs ($12t_2$, 5e, $4t_1$, $3t_1$, $11t_2$, and 4e) are non-bonding combinations of Au 5d AOs. Contours are plotted at ±0.03 a.u; corresponding contributions of atomic orbitals to these MOs are discussed in the main document.

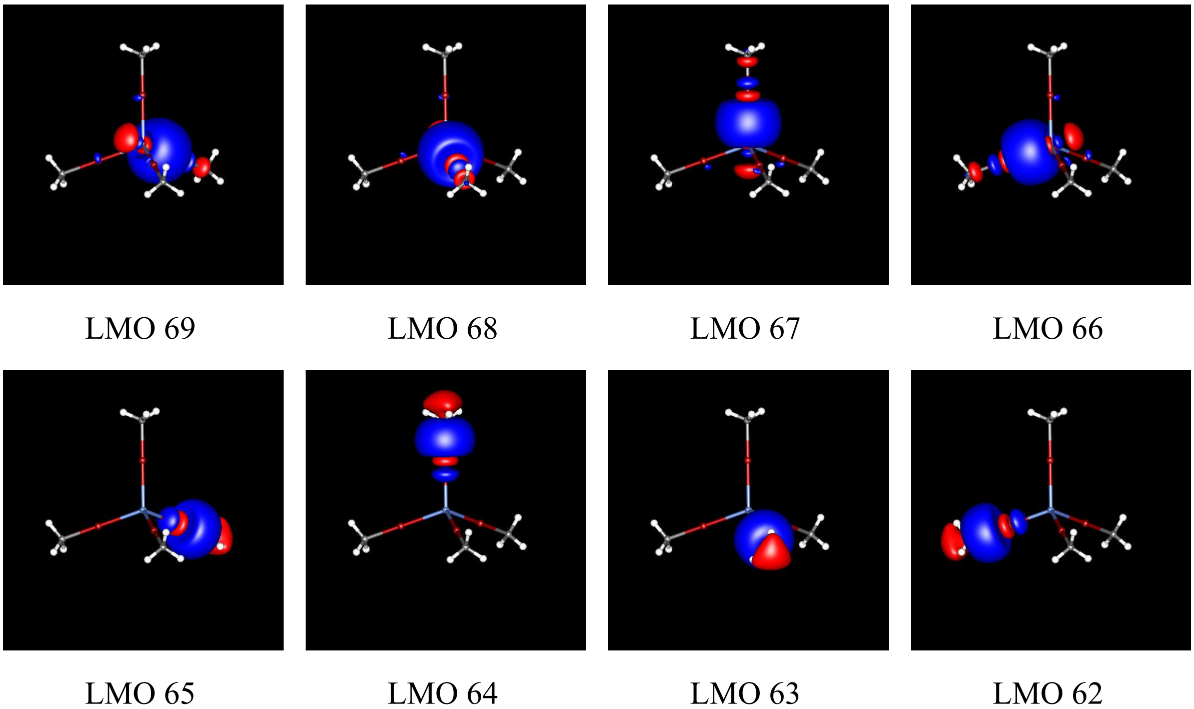

Figure S36. Plots of localized molecular orbitals (LMOs) of the $[Sb(AuMe)_4]^{3-}$ anion in compound **1** representing the four Sb−Au and the four Su−C bonds, with decreasing expectation value of the energy from top left to bottom right. Contours are plotted at ±0.03 a.u.

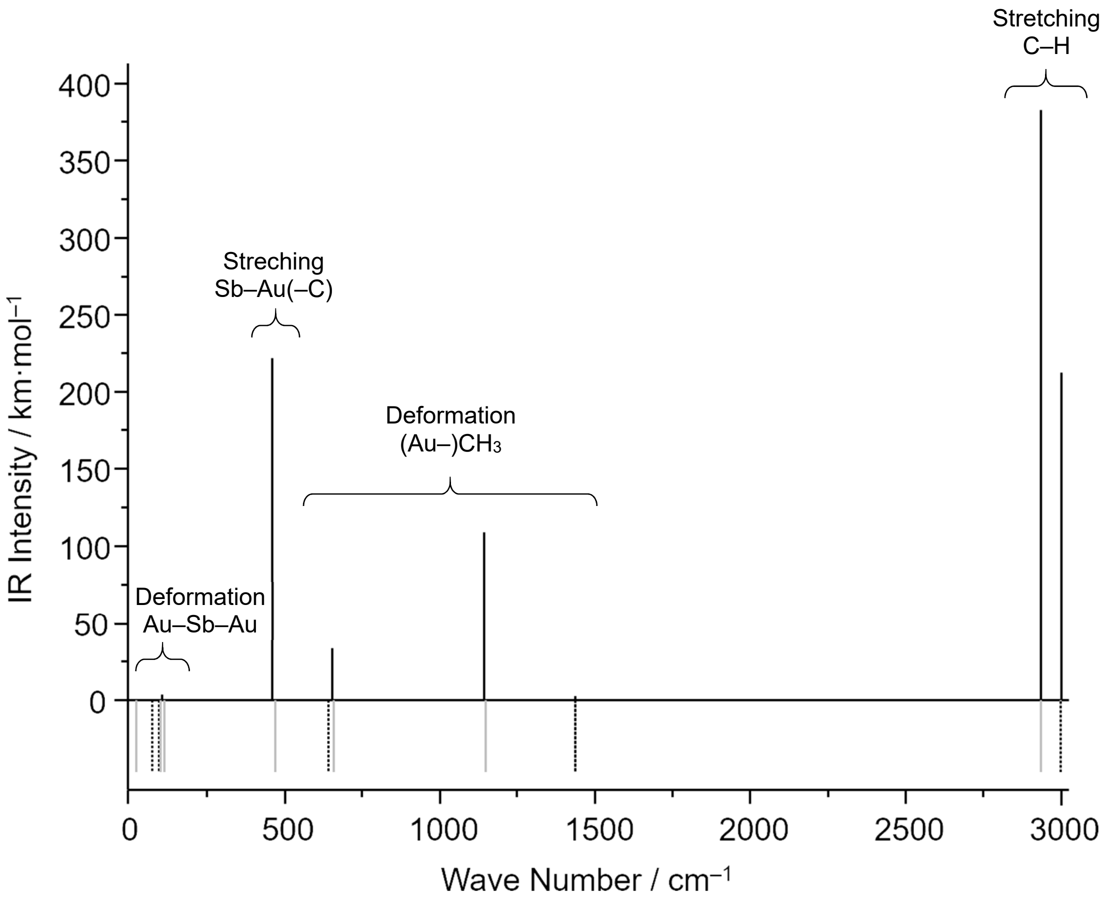

Figure S37. Calculated vibrational spectrum of the anion in **1**. IR modes (with intensities) are drawn upwards in bold black lines. Raman modes are drawn downwards as grey lines without specified intensity. Dashed lines drawn downwards indicate modes that are neither IR-active nor Raman-active for the given molecular symmetry, i.e., no intensity is observed in 1st order, yet possibly upon symmetry breaking during other vibrations. All values and details regarding the calculated modes are given in Table S6.

Table S6. Details of the calculated vibrational modes of the anion in 1. The (arbitrary) intensity value of –5 indicate Raman modes, the intensities of which were not determined. The (arbitrary) intensity value of –1 indicates modes that are neither IR-active nor Raman-active for the given molecular symmetry, i.e., no intensity is observed in 1st order, yet possibly upon symmetry breaking during other vibrations.

Mode #	Symmetry	Wave number cm^{-1}	IR intensity [a] km/mol	Selection rules IR/RAMAN	Characterization of the mode
1	e	22.17	–5.00000	NO/YES	Deformation Au–Sb–Au
2	e	22.17	–5.00000	NO/YES	Deformation Au–Sb–Au
3	t2	24.54	0.05439	YES/YES	Deformation Au–Sb–Au
4	t2	24.54	0.05439	YES/YES	Deformation Au–Sb–Au
5	t2	24.54	0.05439	YES/YES	Deformation Au–Sb–Au
6	t1	75.79	–1.00000	NO/NO	Rotation CH_3 about Au–C bond
7	t1	75.79	–1.00000	NO/NO	Rotation CH_3 about Au–C bond
8	t1	75.79	–1.00000	NO/NO	Rotation CH_3 about Au–C bond
9	a2	76.06	–1.00000	NO/NO	Rotation CH_3 about Au–C bond
10	t1	96.08	–1.00000	NO/NO	Deformation Sb–Au–C
11	t1	96.08	–1.00000	NO/NO	Deformation Sb–Au–C
12	t1	96.08	–1.00000	NO/NO	Deformation Sb–Au–C
13	a1	102.42	–5.00000	NO/YES	Symmetric stretching Sb–Au
14	t2	106.77	3.38350	YES/YES	Deformation Sb–Au–C
15	t2	106.77	3.38350	YES/YES	Deformation Sb–Au–C
16	t2	106.77	3.38350	YES/YES	Deformation Sb–Au–C
17	e	113.28	–5.00000	NO/YES	Deformation Sb–Au–C (+ slightly Au–Sb–Au)
18	e	113.28	–5.00000	NO/YES	Deformation Sb–Au–C (+ slightly Au–Sb–Au)
19	t2	187.15	0.18954	YES/YES	Deformation + breathing Au–Sb(–Au)
20	t2	187.15	0.18954	YES/YES	Deformation + breathing Au–Sb(–Au)
21	t2	187.15	0.18954	YES/YES	Deformation + breathing Au–Sb(–Au)
22	t2	459.83	221.60109	YES/YES	Asymmetric stretching (Sb–)Au–C
23	t2	459.83	221.60109	YES/YES	Asymmetric stretching (Sb–)Au–C
24	t2	459.83	221.60109	YES/YES	Asymmetric stretching (Sb–)Au–C
25	a1	468.58	–5.00000	NO/YES	Symmetric stretching (Sb–)Au–C
26	t1	643.17	–1.00000	NO/NO	Deformation CH_3
27	t1	643.17	–1.00000	NO/NO	Deformation CH_3
28	t1	643.17	–1.00000	NO/NO	Deformation CH_3
29	t2	653.34	33.24035	YES/YES	Deformation CH_3
30	t2	653.34	33.24035	YES/YES	Deformation CH_3
31	t2	653.34	33.24035	YES/YES	Deformation CH_3
32	e	657.23	–5.00000	NO/YES	Deformation (Au–)CH_3
33	e	657.23	–5.00000	NO/YES	Deformation (Au–)CH_3
34	t2	1143.82	109.07427	YES/YES	Deformation CH_3
35	t2	1143.82	109.07427	YES/YES	Deformation CH_3
36	t2	1143.82	109.07427	YES/YES	Deformation CH_3
37	a1	1146.74	–5.00000	NO/YES	Deformation CH_3
38	t1	1436.83	–1.00000	NO/NO	Deformation CH_3
39	t1	1436.83	–1.00000	NO/NO	Deformation CH_3
40	t1	1436.83	–1.00000	NO/NO	Deformation CH_3
41	t2	1437.05	2.12175	YES/YES	Deformation CH_3
42	t2	1437.05	2.12175	YES/YES	Deformation CH_3
43	t2	1437.05	2.12175	YES/YES	Deformation CH_3
44	e	1437.10	–5.00000	NO/YES	Deformation CH_3
45	e	1437.10	–5.00000	NO/YES	Deformation CH_3
46	t2	2937.08	382.46699	YES/YES	Asymmetric stretching C–H
47	t2	2937.08	382.46699	YES/YES	Asymmetric stretching C–H
48	t2	2937.08	382.46699	YES/YES	Asymmetric stretching C–H
49	a1	2937.81	–5.00000	NO/YES	Symmetric stretching C–H
50	e	3001.07	–5.00000	NO/YES	Asymmetric stretching C–H
51	e	3001.07	–5.00000	NO/YES	Asymmetric stretching C–H
52	t1	3001.07	–1.00000	NO/NO	Asymmetric stretching C–H
53	t1	3001.07	–1.00000	NO/NO	Asymmetric stretching C–H
54	t1	3001.07	–1.00000	NO/NO	Asymmetric stretching C–H
55	t2	3001.11	212.13796	YES/YES	Asymmetric stretching C–H
56	t2	3001.11	212.13796	YES/YES	Asymmetric stretching C–H
57	t2	3001.11	212.13796	YES/YES	Asymmetric stretching C–H

6.2 Calculation of anions of the type $[Pn(AuMe)_4]^{3-}$ and cations of the type $[Pn(AuPH_3)_4]^{+}$

All variants of $[Pn(AuMe)_4]^{3-}$ and $[Pn(AuPH_3)_4]^{+}$, Pn = N, P, As, Sb, Bi, in tetrahedral and pyramidal structure were optimized at levels TPSS/dhf-TZVP, HF/dhf-TZVP and SCS-MP2[15]/dhf-TZVPP employing COSMO.[12] Moreover, transition state structures were estimated with a nudge-band type procedure[16] at level TPSS/dhf-TZVP. Further, the structure of $[Sb(AuPH_3)_4]^{+}$ was optimized at SCS-MP2 level with only 16 d(Au) orbitals (see Table S11) being correlated. Coordinates and total energies of all calculated structures are provided in a supplemental ASCII file, 'Structures-Calc.xyz'. As an example, the structures of the Sb-centered molecules optimized at the TPSS/dhf-TZVP level are shown in Figure S38.

Natural charges were calculated by means of natural population analyses (NPA).[17] For Pn = Sb, Mulliken contributions of atomic orbitals to high occupied MOs are listed in Tables S7 to S10 for the optimized tetrahedral and pyramidal structures (level TPSS/dhf-TZVP), and for pyramidal $[Pn(AuPH_3)_4]^{+}$ also at level HF/dhf-TZVP in Table S11.

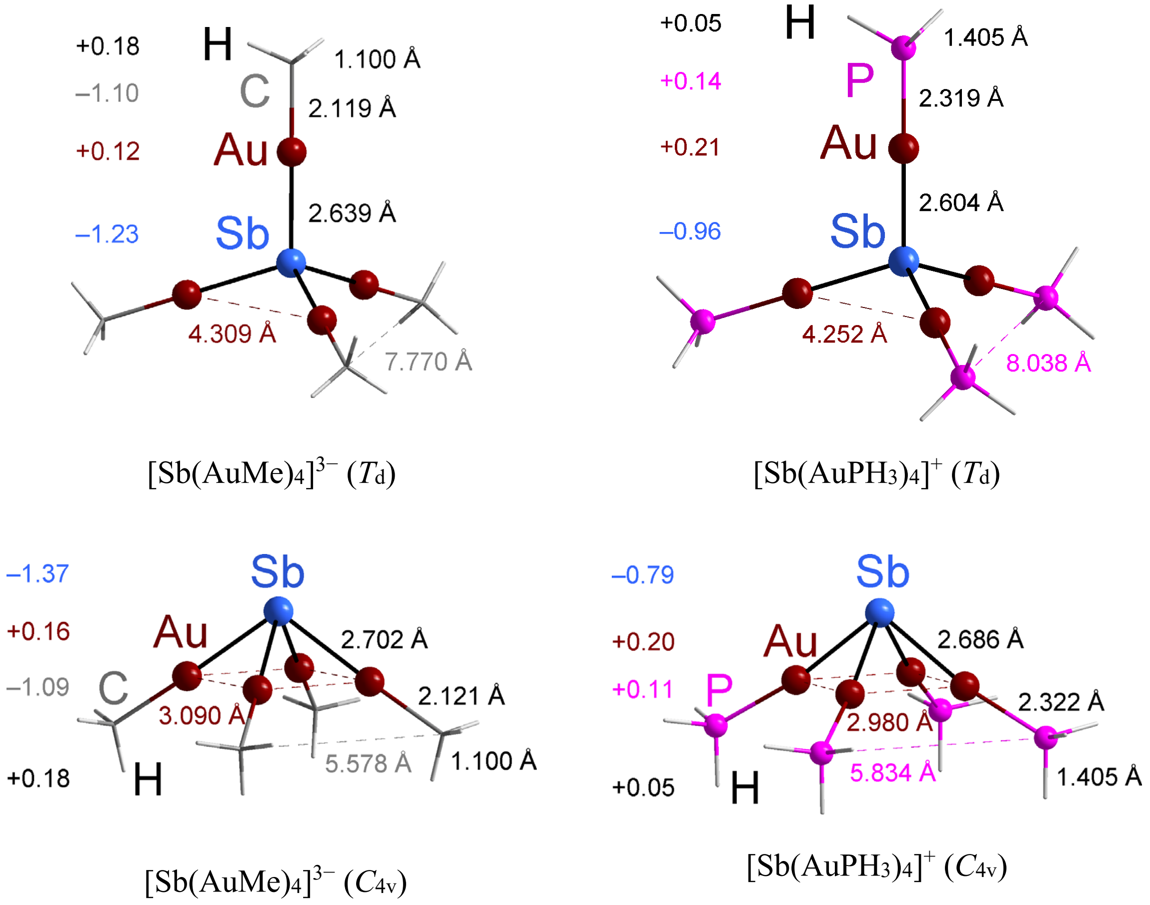

Figure S38. Calculated structures of the anion $[Sb(AuMe)_4]^{3-}$ (left) and the cation $[Sb(AuPH_3)_4]^{+}$ (right), optimized at the TPSS/dhf-TZVP level of theory in T_d symmetry (top) and C_{4v} symmetry (bottom), indicating structural parameters (at the bonds) and natural charges of the atoms (to the left; the color code is that of the atoms).

Table S7. Energies (TPSS/dhf-TZVP) of highest occupied molecular orbitals of tetrahedral $[Sb(AuMe)_4]^{3-}$ and Mulliken contributions of the atomic orbitals in %. T_d symmetry is assumed; the HOMO-LUMO gap amounts to 3.592 eV.

	ε / eV	s(Au)	dAu)	s(Sb)	p(Sb)	s(C)	p(C)	s(H)
13 t_2	–3.915	0	14	0	46	3	28	2
12 t_2	–5.257	51	39	0	1	0	9	0
8 a_1	–5.407	54	24	0	0	1	20	0
4 t_1	–5.803	0	100	0	0	0	0	0
5 e	–5.807	0	100	0	0	0	0	0
3 t_1	–5.833	0	93	0	0	0	2	4
11 t_2	–5.882	1	99	0	0	0	0	0
4 e	–6.041	0	91	0	0	0	2	4
10 t_2	–6.306	2	76	0	11	0	5	4
7 a_1	–7.010	1	48	10	0	1	38	0
9 t_2	–8.076	11	47	0	10	0	26	4
2 t_1	–8.969	0	7	0	0	0	50	43
3 e	–8.972	0	7	0	0	0	50	43
8 t_2	–8.979	0	8	0	0	0	49	42
6 a_1	–12.079	11	10	78	0	0	0	0
7 t_2	–16.101	1	2	0	0	64	1	31
5 a_1	–16.108	2	2	0	0	63	1	31

Table S8. Energies (TPSS/dhf-TZVP) of highest occupied molecular orbitals of pyramidal $[Sb(AuMe)_4]^{3-}$ and Mulliken contributions of the atomic orbitals in %. C_{4v} symmetry is assumed; the HOMO-LUMO gap amounts to 3.042 eV.

	ε / eV	s(Au)	dAu)	s(Sb)	p(Sb)	s(C)	p(C)	s(H)
17 e	–3.744	1	21	0	46	3	22	1
15 a_1	–3.879	1	13	2	57	2	19	0
11 b_2	–4.180	27	44	0	0	3	25	0
16 e	–4.878	30	50	0	0	1	16	0
4 a_2	–5.053	0	100	0	0	0	0	0
10 b_2	–5.387	1	99	0	0	0	0	0
3 a_2	–5.470	0	100	0	0	0	0	0
9 b_2	–5.628	3	96	0	0	0	0	0
15 e	–5.659	0	98	0	0	0	1	0
14 a_1	–5.754	33	50	0	0	0	16	0
14 e	–5.801	9	89	0	2	0	0	0
13 e	–6.096	0	89	0	6	0	4	0
5 b_1	–6.143	0	99	0	0	0	0	0
13 a_1	–6.324	8	87	0	0	0	4	0
12 e	–6.473	12	78	0	2	0	4	2
4 b_1	–6.579	0	89	0	0	0	4	4
12 a_1	–6.722	18	67	0	4	0	4	3
11 a_1	–7.400	4	41	14	10	1	25	4
8 b_2	–7.467	5	52	0	0	1	38	2
11 e	–8.062	14	44	0	11	0	25	5
2 a_2	–8.901	0	6	0	0	0	50	44
7 b_2	–8.924	0	6	0	0	0	50	43
10 e	–8.934	0	8	0	0	0	49	43
9 e	–8.949	0	8	0	0	0	49	42
10 a_1	–8.965	0	9	0	0	0	49	42
3 b_1	–8.966	0	9	0	0	0	49	41
9 a_1	–12.106	12	10	76	1	0	0	0
6 b_2	–16.061	1	2	0	0	63	1	33
8 e	–16.067	1	2	0	0	63	1	32
8 a_1	–16.079	2	2	0	0	62	1	32

Table S9. Energies (TPSS/dhf-TZVP) of highest occupied molecular orbitals of tetrahedral $[Sb(AuPH_3)_4]^+$ and Mulliken contributions of the atomic orbitals in %. T_d symmetry is assumed; the HOMO-LUMO gap amounts to 4.368 eV.

	ε / eV	s(Au)	dAu)	s(Sb)	p(Sb)	s(P)	p(P)	s(H)
16 t_2	–5.797	19	12	0	48	2	10	3
15 t_2	–6.795	29	49	0	0	2	17	2
10 a_1	–6.930	41	48	0	0	1	10	0
5 t_1	–7.181	0	100	0	0	0	0	0
6 e	–7.208	0	100	0	0	0	0	0
14 t_2	–7.248	0	99	0	0	0	0	0
4 t_1	–7.705	0	92	0	0	0	0	4
5 e	–7.863	0	91	0	0	0	0	4
13 t_2	–8.120	0	79	0	10	0	4	4
9 a_1	–9.448	2	23	12	0	2	47	9
12 t_2	–10.386	11	35	0	4	1	35	11
3 t_1	–10.490	0	4	0	0	0	49	45
4 e	–10.492	0	4	0	0	0	48	45
11 t_2	–10.503	1	8	0	1	0	47	42
8 a_1	–13.737	12	13	75	0	0	0	0
10 t_2	–17.085	2	2	0	0	74	1	21
7 a_1	–17.096	2	2	0	0	73	1	22

Table S10. Energies (TPSS/dhf-TZVP) of highest occupied molecular orbitals of pyramidal $[Sb(AuPH_3)_4]^+$ and Mulliken contributions of the atomic orbitals in %. C_{4v} symmetry is assumed; the HOMO-LUMO gap amounts to 3.400 eV.

	ε / eV	s(Au)	dAu)	s(Sb)	p(Sb)	s(P)	p(P)	s(H)
21 e	–5.499	15	21	0	41	2	10	2
18 a_1	–5.817	9	12	9	49	2	11	0
14 b_2	–6.142	18	61	0	0	3	18	0
20 e	–6.760	20	60	0	0	2	16	0
5 a_2	–6.801	0	100	0	0	0	0	0
13 b_2	–7.014	3	97	0	0	0	0	0
4 a_2	–7.305	0	97	0	0	0	0	0
19 e	–7.344	0	97	0	1	0	1	0
18 e	–7.512	9	88	0	0	0	1	0
17 a_1	–7.513	25	64	0	0	1	9	0
12 b_2	–7.597	1	93	0	0	0	0	2
6 b_1	–7.864	0	100	0	0	0	0	0
17 e	–8.115	0	83	0	8	0	3	3
16 a_1	–8.121	16	81	0	0	0	3	0
16 e	–8.428	6	81	0	2	0	4	3
15 a_1	–8.574	7	75	0	3	0	5	4
5 b_1	–8.693	0	89	0	0	0	2	5
14 a_1	–9.803	5	27	18	3	1	35	9
11 b_2	–10.001	7	37	0	0	1	43	10
15 e	–10.371	7	22	0	3	0	40	24
3 a_2	–10.434	0	4	0	0	0	49	45
10 b_2	–10.446	0	4	0	0	0	49	45
14 e	–10.458	0	5	0	0	0	48	44
13 a_1	–10.483	0	8	0	0	0	47	43
4 b_1	–10.490	0	8	0	0	0	47	42
13 e	–10.517	5	22	0	3	0	40	28
12 a_1	–13.830	13	13	71	2	0	0	0
9 b_2	–17.047	0	2	0	0	74	1	22
12 e	–17.054	1	2	0	0	74	1	21
11 a_1	–17.073	3	2	0	0	73	1	21

Table S11. Hartree-Fock orbital energies of highest occupied molecular orbitals of pyramidal $[Sb(AuPH_3)_4]^+$ and Mulliken contributions of the atomic orbitals in %. C_{4v} symmetry is assumed. The structure parameters result from an optimization at level SCS-MP2/dhf-TZVPP where only the orbitals with dominant Au(d) contributions (i.e. those with energies between -13.3 and -10.5 eV) were included in the MP2 treatment. See also main document.

	ε / eV	s(Au)	dAu)	s(Sb)	p(Sb)	s(P)	p(P)	s(H)
18 a1	–7.334	8	4	5	70	1	5	0
21 e	–7.682	21	6	0	57	1	5	0
14 b2	–9.911	20	42	0	0	5	33	0
20 e	–10.391	23	34	0	0	5	34	4
17 a1	–10.956	34	32	0	0	4	29	0
5 a2	–11.975	0	**100**	0	0	0	0	0
13 b2	–12.205	0	**100**	0	0	0	0	0
4 a2	–12.329	0	**100**	0	0	0	0	0
19 e	–12.369	1	**98**	0	1	0	0	0
12 b2	–12.404	0	**97**	0	0	0	1	0
18 e	–12.444	0	**100**	0	0	0	0	0
17 e	–12.702	0	**96**	0	1	0	2	0
6 b1	–12.781	0	**100**	0	0	0	0	0
16 a1	–12.831	1	**99**	0	0	0	0	0
16 e	–13.017	1	**95**	0	1	0	2	0
15 a1	–13.144	0	**89**	0	3	0	3	3
5 b1	–13.244	0	**97**	0	0	0	1	0
14 a1	–13.383	1	44	15	2	2	29	4
11 b2	–13.919	1	55	0	0	2	34	6
15 e	–14.330	2	57	0	5	1	27	5
10 b2	–15.350	0	4	0	0	0	45	47
14 e	–15.356	0	4	0	0	0	45	47
3 a2	–15.357	0	3	0	0	0	45	48
13 a1	–15.370	0	5	0	0	0	44	47
13 e	–15.379	0	5	0	0	0	44	47
4 b1	–15.391	0	6	0	0	0	44	47
12 a1	–18.138	8	16	73	1	0	0	0
9 b2	–24.085	0	0	0	0	67	1	32
12 e	–24.088	1	1	0	0	65	1	32
11 a1	–24.094	0	0	0	0	67	1	32

6.3 Investigations of hypothetical formation reactions of the $[Sb(AuMe)_4]^{3-}$ anion in 1, the[$(Sn_2Sb_2)_2Au]^{3-}$ anion in A, and the $[(Sn_5Sb_3Au)_2]^{4-}$ anion in B

Table S12. Total energies E_{tot} of calculated species used (together with the listed atomization energies) to calculate the reaction energies for the reactions indicated in Scheme 2 in the main document and in Table S13.

Species	E_{tot} [E_h]
Sn	–214.1649289151
Sb	–240.1171224363
$(Sn_2Sb_2)^{2-}$	–909.1572122542
$[AuCH_3PPh_3]$	–1212.406030397
$[Sb(AuMe)_4]^{3-}$	–943. 2633702230
PPh_3	–1036.718517502
$[(Sn_5Sb_3Au)_2]^{4-}$	–3856.013752180
$[(Sn_2Sb_2)_2Au]^{3-}$	–1953.992243495
$H_2NCH_2CH_2NH_2$ (en)	–190.6326396747
$(H_2NCH_2CH_2NH)^-$	–190.0861837792
$[AuMe_2]^-$	–215.7021125101
CH_4	–40.54093502810
C_5H_5N (py)	–248.4275209928
$C_{10}H_8N_2$ (4,4'-bipy) [a]	–495.6558882561
$(C_{10}H_8N_2)^{\bullet-}$ (4,4'-bipy-anion radical)	–495.7500349461
$(C_{19}H_8N_2)^{2-}$ (4,4'-bipy-dianion)	–495.8150762125
$C_{10}H_8N_2$ (2,2'-bipy) [a]	–495.6613238796
$(C_{10}H_8N_2)^{\bullet-}$ (2,2'-bipy-anion radical) [a]	–495.7434216528
$(C_{19}H_8N_2)^{2-}$ (2,2'-bipy-dianion) [a]	–495.7978441189
H_2	–1.180054681885
	Atomization Energy[15] [kJ/mol]
Sn	302
Sb	262

[a] These species (indicated in grey) were calculated for comparison, yet were not taken into consideration for calculating reaction energies.

Table S13. Hypothetical reaction schemes for the formation of the anions in compounds A, B, and 1, with increasing amount of [AuMePPh$_3$] in solvents py or en. Reaction energies are calculated based on the total energies of the species highlighted in grey in Table S6 (1 E$_h$ = 2625.47 kJ/mol). Most exoenergetic reactions per type are highlighted in grey.

Reaction type		ΔE [kJ/mol]
A	Ratio $(Sn_2Sb_2)^{2-}$: [AuMePPh$_3$] = 5 : 2	
*A1	$(CH_3)^-$ "caught" by py under formation of (4,4'-bipy)$^{\bullet-}$ and CH_4 and H_2 [a]	
	5 $(Sn_2Sb_2)^{2-}$ + 2 [AuMePPh$_3$] + 4 C_5H_5N ⟶ $[(Sn_5Sb_3Au)_2]^{4-}$ + 2 PPh_3 + 2 $(C_{10}H_8N_2)^{\bullet-}$ + 2 CH_4 + H_2 + 4/3 "Sb^{3-}" + 8/3 Sb^0	N/A
*A2	$(CH_3)^-$ "caught" by py under formation of (4,4'-bipy)$^{2-}$ and CH_4 [a]	
	5 $(Sn_2Sb_2)^{2-}$ + 2 [AuMePPh$_3$] + 2 C_5H_5N ⟶ $[(Sn_5Sb_3Au)_2]^{4-}$ + 2 PPh_3 + $(C_{10}H_8N_2)^{2-}$ + 2 CH_4 + 4/3 "Sb^{3-}" + 8/3 Sb^0	N/A
A3	$(CH_3)^-$ and additional e^- "caught" by py under formation of (4,4'-bipy)$^{\bullet-}$, CH_4, and H_2 [a]	
	5 $(Sn_2Sb_2)^{2-}$ + 2 [AuMePPh$_3$] + 12 C_5H_5N ⟶ $[(Sn_5Sb_3Au)_2]^{4-}$ + 2 PPh_3 + 6 $(C_{10}H_8N_2)^{\bullet-}$ + 2 CH_4 + 5 H_2 + 4 Sb^0	–190.14 [b] (+857.86)
A4	$(CH_3)^-$ and additional e^- "caught" by py under formation of (4,4'-bipy)$^{2-}$, CH_4, and H_2 [a]	
	5 $(Sn_2Sb_2)^{2-}$ + 2 [AuMePPh$_3$] + 6 C_5H_5N ⟶ $[(Sn_5Sb_3Au)_2]^{4-}$ + 2 PPh_3 + 3 $(C_{10}H_8N_2)^{2-}$ + 2 CH_4 + 2 H_2 + 4 Sb^0	–111.33 [b] (+936.37)
*A5	$(CH_3)^-$ "caught" by $H_2NCH_2CH_2NH_2$ under formation of $(H_2NCH_2CH_2NH)^-$ and CH_4	
	5 $(Sn_2Sb_2)^{2-}$ + 2 [AuMePPh$_3$] + 2 $H_2NCH_2CH_2NH_2$ ⟶ $[(Sn_5Sb_3Au)_2]^{4-}$ + 2 PPh_3 + 2 $(H_2NCH_2CH_2NH)^-$ + 2 CH_4 + 4/3 "Sb^{3-}" + 8/3 Sb^0	N/A
B	Ratio $(Sn_2Sb_2)^{2-}$: [AuMePPh$_3$] = 2 : 1	
B1	$(CH_3)^-$ "caught" by py under formation of (4,4'-bipy)$^{\bullet-}$ and CH_4 and H_2 [a]	
	2 $(Sn_2Sb_2)^{2-}$ + [AuMePPh$_3$] + 2 C_5H_5N ⟶ $[(Sn_2Sb_2)_2Au)]^{3-}$ + PPh_3 + $(C_{10}H_8N_2)^{\bullet-}$ + CH_4 + 0.5 H_2	–42.69
B2	$(CH_3)^-$ "caught" by py under formation of (4,4'-bipy)$^{2-}$ and CH_4 [a]	
	2 $(Sn_2Sb_2)^{2-}$ + [AuMePPh$_3$] + C_5H_5N ⟶ $[(Sn_2Sb_2)_2Au)]^{3-}$ + PPh_3 + 0.5 $(C_{10}H_8N_2)^{2-}$ + CH_4	–29,56
B3	$(CH_3)^-$ "caught" by $H_2NCH_2CH_2NH_2$ under formation of $(H_2NCH_2CH_2NH)^-$ and CH_4	
	2 $(Sn_2Sb_2)^{2-}$ + [AuMePPh$_3$] + $H_2NCH_2CH_2NH_2$ ⟶ $[(Sn_2Sb_2)_2Au)]^{3-}$ + PPh_3 + $(H_2NCH_2CH_2NH)^-$ + CH_4	+39.95
C	Ratio $(Sn_2Sb_2)^{2-}$: [AuMePPh$_3$] = 5 : 4	
*C1	$(CH_3)^-$ used to form $[AuMe_2]^-$	
	5 $(Sn_2Sb_2)^{2-}$ + 4 [AuMePPh$_3$] ⟶ $[(Sn_5Sb_3Au)_2]^{4-}$ + 2 $[AuMe_2]^-$ + 4 PPh_3 + 4/3 "Sb^{3-}" + 8/3 Sb^0	N/A
D	Ratio $(Sn_2Sb_2)^{2-}$: [AuMePPh$_3$] = 1 : 2	
D1	$(CH_3)^-$ "caught" by py under formation of (4,4-bipy)$^{\bullet-}$ and CH_4 and H_2 [a]	
	6 $(Sn_2Sb_2)^{2-}$ + 10 [AuMePPh$_3$] + 4 C_5H_5N ⟶ $[(Sn_5Sb_3Au)_2]^{4-}$ + 2 $[Sb(AuMe)_4]^{3-}$ + 10 PPh_3 + 2 Sn^0 + 4 Sb^0 + 2 $(C_{10}H_8N_2)^{\bullet-}$ + 2 CH_4 + H_2	–529.68 [b] (+1122.32)
D2	$(CH_3)^-$ used to form $[AuMe_2]^-$	
	6 $(Sn_2Sb_2)^{2-}$ + 12 [AuMePPh$_3$] ⟶ $[(Sn_5Sb_3Au)_2]^{4-}$ + 2 $[Sb(AuMe)_4]^{3-}$ + 2 $[AuMe_2]^-$ + 12 PPh_3 + 2 Sn^0 + 4 Sb^0	–470.05 [b] (+1181.95)

Table S13 (continued)

D3	$(CH_3)^-$ "caught" by py under formation of $(4,4\text{-bipy})^{2-}$ and CH_4 [a]	
	$6\ (Sn_2Sb_2)^{2-} + 10\ [AuMePPh_3] + 2\ C_5H_5N$ $\longrightarrow [(Sn_5Sb_3Au)_2]^{4-} + 10\ PPh_3 + 2\ Sn^0 + 4\ Sb^0 + (C_{10}H_8N_2)^{2-} + 2\ CH_4 + 2\ [Sb(AuMe)_4]^{3-}$	–503.41 [b] (+1148.59)
D4	$(CH_3)^-$ "caught" by $H_2NCH_2CH_2NH_2$ under formation of $(H_2NCH_2CH_2NH)^-$ and CH_4	
	$6\ (Sn_2Sb_2)^{2-} + 10\ [AuMePPh_3] + 2\ H_2NCH_2CH_2NH_2$ $\longrightarrow [(Sn_5Sb_3Au)_2]^{4-} + 10\ PPh_3 + 2\ Sn^0 + 4\ Sb^0 + 2\ (H_2NCH_2CH_2NH)^- + 2\ CH_4 + 2\ [Sb(AuMe)_4]^{3-}$	–364.40[b] (+1287.60)
E	Ratio $(Sn_2Sb_2)^{2-}$: $[AuMePPh_3]$ = 3 : 8	
E1	$3\ (Sn_2Sb_2)^{2-} + 8\ [AuMePPh_3]$ $\longrightarrow 2\ [Sb(AuMe)_4]^{3-} + 8\ PPh_3 + 6\ Sn^0 + 4\ Sb^0$	–269.29 (+2590.71)

* Reaction energies for reactions indicated with an asterisk cannot be calculated, as the atomic anion "Sb^{3-}" cannot be reasonably modeled by means of quantum chemistry. [a] Note that the formation of the energetically preferred 4,4'-isomers of $(bipy)^{\bullet-}$ and $(bipy)^{2-}$ are considered only. [b] Values were corrected by consideration of atomization energies to account for the instant precipitation of the metal "atoms" in condensed phase (uncorrected values are given in parentheses).

The correction of data by experimentally observed atomization energies[18] for all reactions that produce neutral atoms is needed (although representing a mixture of methods) to account for the fact that in condensed phases, the atoms would never stay isolated, but would instantly form metal precipitates; this is in accordance with our observations. Furthermore, in most of the directly compared reactions, the amount of metal atoms that form according to the reaction schemes is the same, so the errors are systematically the same, too, in turn enabling the direct comparison.

We note that the indicated side-products, PPh_3, CH_4, H_2, $[AuMe_2]^-$,[19] and the metals Sn and Sb were experimentally proven, thus generally rationalizing the reaction schemes (see Section 5 for details).

The detection of $(H_2NCH_2CH_2NH)^-$ in en solution has not been possible to date, as the signals are covered by those of neutral en and crypt-222, and ESI mass spectrometry would not serve to distinguish the anion's abundance in solution from its formation during the electrospray-ionization process. However, the formation of this anion was suggested in several formation reactions of Zintl clusters in previous works, as the best possible suggestion.[20]

Bipyridine anions, $(C_{10}H_8N_2)^{\bullet-}$ and $(C_{10}H_8N_2)^{2-}$, which were suggested by Sevov and co-authors to form during the formation of the heterometallic cluster anion $[Bi_4Ni_4(CO)_6]^{2-}$ from $[Bi_3Ni_4(CO)_6]^{3-}$, and by our group in the context of the formation of Bi_{11}^{3-} from $(GaBi_3)^{2-}$ in pyridine,[21] cannot be reasonably detected in the reaction mixture, as ^{1}H-NMR is not indicative for it besides py and PPh_3, as EPR fails in Zintl chemistry reaction mixtures (in contrast to solutions of isolated products), and as ESI MS would not work for the same reasons as mentioned for the $(H_2NCH_2CH_2NH)^-$ anion. However, its formation was previously proven by corresponding crystal structures.[22] Also in the context of Zintl chemistry, the Sevov group reported that, upon dissolving K_5Bi_4 in pyridine in the presence of crypt-222, a dark-brown solution is formed. After stirring for several hours (4 h and more), the color turned to dark purple, indicative of the presence of $(C_{10}H_8N_2)^{\bullet-}$.[23]

7 References for the Supporting Information

[1] 4,7,13,16,21,24-Hexaoxa-1,10-diazabicyclo[8.8.8]hexacosane.
[2] F. Lips, I. Schellenberg, R. Pöttgen, S. Dehnen, *Chem. Eur. J.* **2009**, *15*, 12968–12973.
[3] F.-X. Pan, L. J. Li, Z. M. Sun, *Chin. J. Struct. Chem.* **2016**, *35*, 1099−1106.
[4] a) S. M. Sheldrick, *Acta Crystallogr* **2015**, *A71*, 3; b) G. M. Sheldrick, *Acta Crystallogr.* **2015**, *C71*, 3.
[5] a) P. van der Sluis, A. L. Spek, *Acta Crystallogr.* **1990**, *A46*, 194; b) A. Spek, *Acta Crystallogr.* **2009**, *D65*, 148.
[6] Diamond - Crystal and Molecular Structure Visualization, Crystal Impact - Dr. H. Putz & Dr. K. Brandenburg GbR, Kreuzherrenstr. 102, 53227 Bonn, Germany, http://www.crystalimpact.com/diamond.
[7] Z. Hassanzadeh Fard, C. Müller, T. Harmening, R. Pöttgen, S. Dehnen, *Angew. Chem.* **2009**, *121*, 4507–4511; *Angew. Chem. Int. Ed.* **2009**, *48*, 4441–4444.
[8] a) TURBOMOLE V7.5.1 2021, a development of University of Karlsruhe and Forschungszentrum Karlsruhe GmbH, 1989–2007, TURBOMOLE GmbH, since 2007; available from http://www.turbomole.com; b) S. G. Balasubramani, G. P. Chen, S. Coriani, M. Diedenhofen, M. S. Frank, Y. Franzke, F. Furche, R. Grotjahn, M. Harding, C. Hättig, A. Hellweg, B. Helmich-Paris, C. Holzer, U. Huniar, M. Kaupp, A. M. Khah, S. K. Khani, T. Müller, F. Mack, B. D. Nguyen, S. Parker, W. Perlt, D. Rappoport, K. Reiter, S. Roy, M. Rückert, G. Schmitz, M. Sierka, E. Tapavicza, D. Tew, P. C. van Wüllen, V. K. Voora, F. Weigend, A. Wodyński, J. M. Yu, *J. Chem. Phys.* **2020**, *152*, 184107.
[9] J. Tao, J. P. Perdew, V. N. Staroverov, G. E. Scuseria, *Phys. Rev. Lett.* **2003**, *91*, 146401.
[10] a) F. Weigend, R. Ahlrichs, *Phys. Chem. Chem. Phys.* **2005**, *7*, 3297–3305; b) F. Weigend, A. Baldes, *J. Chem. Phys.* **2010**, *133*, 174102.
[11] a) B. Metz, H. Stoll, M. Dolg, *J. Chem. Phys.* **2000**, *113*, 2563; b) D. Figgen, G. Rauhaut, M. Dolg, H. Stoll, *Chem. Phys.* **2005**, *311*, 227–244.
[12] A. Klamt, G. Schüürmann, *J. Chem. Soc., Perkin Trans. 2* **1993**, 799–805.
[13] S. F. Boys, in *Quantum Theory of Atoms, Molecules and the Solid State* (Ed.: P.-O. Löwdin), New York, **1966**, 253–262.
[14] a) L. Laaksonen, *J. Mol. Graph.* **1992**, *10*, 33–34; b) D. L. Bergman, L. Laaksonen, A. Laaksonen, *J. Mol. Graph. Model.* **1997**, *15*, 301–306.
[15] S.Grimme, *J. Chem. Phys.* **2003**, *118*, 9095-9102.
[16] P. Plessow, *J. Chem. Theory Comput.* **2013**, 9, 1305–1310.
[17] A. E. Reed, R. B. Weinstock, F. Weinhold, *J. Chem. Phys.* **1985**, *83*, 735–746.
[18] J. D. Cox, D. D. Wagman, and V. A. Medvedev, *CODATA Key Values for Thermodynamics, Hemisphere Publishing Corp.*, New York, USA, **1989**.
[19] a) A. Tamaki, J. K. Kochi, *J. Organornet. Chem.* **1973**, 51, C39–C42; b) G. W. Rice, R. S. Tobias, *Inorg. C'hem.* **1976**, *15*, 4–9; c) R. J. Puddephatt, *Gold Bulletin* **1977**, *10*, 108–113; d) D. Zhu, S. V. Lindeman, J. K. Kochi, *Organometallics* **1999**, *18*, 2241–2248.
[20] a) M. W. Hull, C. Sevov, *J. Am. Chem. Soc.* **2009**, *131*, 9026–9037; b) F. Lips, R. Clérac, S. Dehnen, *J. Am. Chem. Soc.* **2011**, *133*, 14168–14171; c) B. Weinert, F. Müller, K. Harms, R. Clérac, S. Dehnen, *Angew. Chem. Int. Ed.* **2014**, *53*, 11979–11983; d) A. R. Eulenstein, Y. J. Franzke, N. Lichtenberger, R. J. Wilson, L. Deubner, F. Kraus, R. Clérac, F. Weigend, S. Dehnen, *Nat. Chem.* **2021**, *13*, 149–155.
[21] a) J. M. Goicoechea, M. W. Hull, S. C. Sevov, *J. Am. Chem. Soc.* **2007**, *129*, 7885–7893; b) B. Weinert, A. R. Eulenstein, R. Ababei, S. Dehnen, *Angew. Chem.* **2014**, *126*, 4792–4797; *Angew. Chem. Int. Ed.* **2014**, *53*, 4704–4708.
[22] a) M. S. Denning, M. Irwin, J. M. Goicoechea, *Inorg. Chem.* **2008**, *47*, 6118–6120; b) J. Schröder, D. Himmel, D. Kratzert, V. Radtke, S. Richert, S. Weber, T. Böttcher, *Chem. Commun.* **2019**, *55*, 1322–1325.
[23] L. G. Perla, A. G. Oliver, S. C. Sevov, *Inorg. Chem.* **2015**, *54*, 872–875.
